# Assessing the reliability of spike-in normalization for analyses of single-cell RNA sequencing data

**DOI:** 10.1101/gr.222877.117

**Published:** 2017-11

**Authors:** Aaron T.L. Lun, Fernando J. Calero-Nieto, Liora Haim-Vilmovsky, Berthold Göttgens, John C. Marioni

**Affiliations:** 1 Cancer Research UK Cambridge Institute, University of Cambridge, Li Ka Shing Centre, Cambridge CB2 0RE, United Kingdom;; 2 Wellcome Trust and MRC Cambridge Stem Cell Institute, University of Cambridge, Cambridge CB2 0XY, United Kingdom;; 3 EMBL European Bioinformatics Institute, Wellcome Genome Campus, Hinxton, Cambridge CB10 1SD, United Kingdom;; 4 Wellcome Trust Sanger Institute, Wellcome Genome Campus, Hinxton, Cambridge CB10 1SA, United Kingdom

## Abstract

By profiling the transcriptomes of individual cells, single-cell RNA sequencing provides unparalleled resolution to study cellular heterogeneity. However, this comes at the cost of high technical noise, including cell-specific biases in capture efficiency and library generation. One strategy for removing these biases is to add a constant amount of spike-in RNA to each cell and to scale the observed expression values so that the coverage of spike-in transcripts is constant across cells. This approach has previously been criticized as its accuracy depends on the precise addition of spike-in RNA to each sample. Here, we perform mixture experiments using two different sets of spike-in RNA to quantify the variance in the amount of spike-in RNA added to each well in a plate-based protocol. We also obtain an upper bound on the variance due to differences in behavior between the two spike-in sets. We demonstrate that both factors are small contributors to the total technical variance and have only minor effects on downstream analyses, such as detection of highly variable genes and clustering. Our results suggest that scaling normalization using spike-in transcripts is reliable enough for routine use in single-cell RNA sequencing data analyses.

Single-cell RNA sequencing (scRNA-seq) is a powerful technique for studying transcriptional activity in individual cells. Briefly, RNA is isolated from single cells, reverse transcribed into cDNA, and sequenced using massively parallel sequencing technologies (Shapiro et al. 2013). This can be performed using microfluidics platforms like the Fluidigm C1 (Pollen et al. 2014), with protocols such as Smart-seq2 (Picelli et al. 2014) that use microtiter plates; or with droplet-based technologies (Klein et al. 2015; Macosko et al. 2015) that can profile thousands of cells. Gene expression is quantified by mapping read sequences to a reference genome and counting the number of reads mapped to each annotated gene. To avoid amplification biases, individual transcript molecules can also be tagged with unique molecular identifiers (UMIs) (Islam et al. 2014), such that sequencing to saturation and counting UMIs will yield the number of transcripts of each gene in a cell. Regardless of whether reads or UMIs are used, not all transcript molecules will be captured and sequenced due to cell-specific inefficiencies in reverse transcription (Stegle et al. 2015). The presence of these cell-specific biases compromises the direct use of the read/UMI count as a quantitative measure of gene expression. Normalization is required to remove these biases before the gene counts can be meaningfully compared between cells in downstream analyses.

A common normalization strategy for RNA-seq data uses a set of genes that have constant expression across cells. This set can consist of predefined “housekeeping” genes, or it can be empirically defined under the assumption that most genes are not differentially expressed (DE) between cells (Anders and Huber 2010; Robinson and Oshlack 2010; Lun et al. 2016a). Any systematic differences in expression between cells for this non-DE set of genes must, therefore, be technical in origin, e.g., due to differences in library size or composition bias (Robinson and Oshlack 2010). Counts are scaled to eliminate these differences, yielding normalized expression values for downstream analyses. This gene-based approach works well for bulk sequencing experiments in which the population-wide gene expression profile is stable. However, it may not be suitable for single-cell experiments in which strong biological heterogeneity complicates the identification of a reliable non-DE set. For example, housekeeping genes may be turned on or off by transcriptional bursting, whereas processes like the cell cycle may trigger large-scale changes in the expression profile that preclude a non-DE majority.

An alternative normalization approach is to use spike-in RNA for which the identity and quantity of all transcripts is known (Stegle et al. 2015; Bacher and Kendziorski 2016). The same amount of spike-in RNA is added to each cell's lysate, and the spike-in transcripts are processed in parallel with their endogenous counterparts to generate a sequencing library. This yields a set of read (or UMI) counts for both endogenous and spike-in transcripts in each cell. Normalization is performed by scaling the counts for each cell such that the counts for the spike-in genes are, on average, the same between cells (Katayama et al. 2013). The central assumptions of this approach are that (1) the same amount of spike-in RNA is added to each cell; and (2) the spike-in and endogenous transcripts are similarly affected by cell-to-cell fluctuations in capture efficiency. Under these assumptions, any differences in the coverage of the spike-in transcripts between cells must be artifactual in origin and should be removed by scaling. One particular advantage of this strategy is that it does not make any assumptions about the endogenous expression profile, unlike the non-DE approach described above. This means that spike-in normalization can be applied in situations in which large-scale changes in expression (e.g., related to changes in total RNA content, or involving highly heterogeneous populations containing many cell types) are expected and of interest (Lun et al. 2016b; Nestorowa et al. 2016).

There are two common criticisms of spike-in normalization that challenge the validity of its central assumptions. The first is that the same quantity of spike-in RNA may not be consistently added to each sample (Robinson and Oshlack 2010), and the second is that synthetic spike-in transcripts may not behave in the same manner as endogenous transcripts (Grün and van Oudenaarden 2015) (i.e., the two sets of transcripts have unequal capture efficiencies, caused by differences in their biophysical properties). Any differences in spike-in quantity or behavior across cells will compromise the accuracy of spike-in normalization (Risso et al. 2014). In some cases, it may also be difficult to gauge how much spike-in RNA should be added, especially if the quantity of endogenous RNA per cell is unknown, resulting in insufficient spike-in coverage for normalization. These criticisms may contribute to the limited use of this normalization strategy in the scRNA-seq literature (Bacher and Kendziorski 2016). However, if one were to dismiss the use of spike-in normalization, there would be no general alternative for removing cell-specific biases in scRNA-seq data sets where a non-DE majority of genes cannot be assumed. Thus, it is of particular interest whether or not the aforementioned criticisms of spike-in normalization are relevant to real scRNA-seq experiments. To our knowledge, this has yet to be rigorously studied.

## Results

### Overview

In this paper, we conduct a series of experiments to estimate the reliability of spike-in normalization in single-cell transcriptome studies using plate-based protocols. We use mixtures of two distinct spike-in RNA sets to quantify the variance of the added spike-in volume across cells and show that it is quantitatively negligible in real experiments across a range of conditions. We also obtain an upper bound on the cell-to-cell variability in the differences in behavior (i.e., the fold changes in the capture efficiencies) between the two spike-in sets. Simulations indicate that both factors have only minor effects on the results of downstream analyses, such as detection of DE and highly variable genes. These results suggest that spike-ins can be safely used for routine normalization of scRNA-seq data.

We emphasize that we are only interested in the performance of spike-in RNA for scaling normalization. This involves the calculation of cell-specific scaling factors to remove relative biases between cells. We are not investigating the performance of spike-in RNA for the absolute quantification of endogenous transcript molecules (Svensson et al. 2017), which would require estimation of the absolute bias in each cell. We are also not studying the use of spike-ins for batch correction (Tung et al. 2017), which would require modeling of gene-specific batch effects beyond simple cell-specific scaling. Both of these tasks are separate to scaling normalization and will not be addressed here.

### Description of the mixture experiments

**Figure 1.**
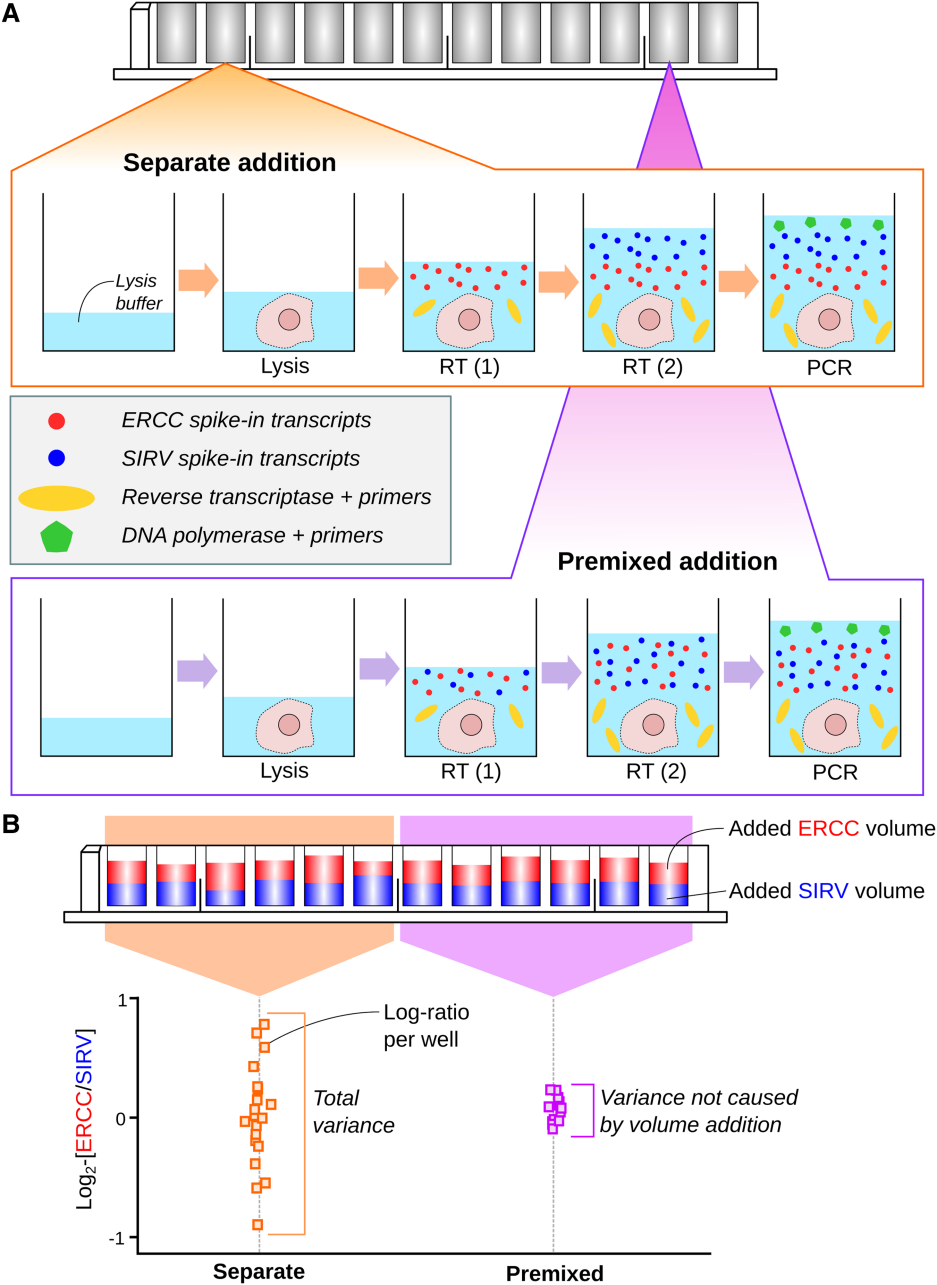
Schematic of the experimental design to assess the variability of spike-in addition in a plate-based scRNA-seq protocol. (*A*) A cell is sorted into each well of a plate and lysed. For one set of wells, an equal volume of each spike-in set is added separately, along with the reverse transcription (RT) reagents. For another set of wells, an equal volume of a pooled mixture of the two spike-ins is added into each well (done twice to keep the protocol consistent). Reverse transcription, PCR amplification, library generation, and sequencing were then performed. (*B*) The log_2_-ratio between the total counts of the two spike-in sets was computed for each well. The variance of the log-ratio was estimated from all wells with separate addition of spike-ins and from wells with addition of the premixed pool. The difference between these two estimates represents the variance attributable to volume addition.

We aimed to assess the variability in the added spike-in quantity across cells. To do so, we performed mixture experiments using two distinct spike-in sets (Fig. 1)—the External RNA Controls Consortium (ERCC) set and the Spike-in RNA Variants (SIRV) set. An equal volume of each spike-in set was added separately to all wells of a 96-well microtiter plate. Each well contained a single lysed mouse cell—a mouse 416B myeloid progenitor cell or trophoblast stem cell (TSC)—thus mimicking real experimental conditions. The resulting pool of endogenous/spike-in RNA in each well was used to generate a cDNA library, using a modified version of the Smart-seq2 protocol (Methods). This process was repeated for all wells, and high-throughput sequencing was performed on all libraries.

For each library, reads were mapped to the genome and assigned to genes to quantify expression. The total count was computed across all transcripts of each spike-in set in each well. The log_2_-ratio of the totals between the two sets was computed for each well, and the variance of this log-ratio was computed across wells. Any variability in spike-in volume addition should manifest as an increase in the variability of the log-ratio, given that the spike-in sets were added independently to each well.

We also repeated the experiment by adding volumes of “premixed” spike-in solution where the two spike-in sets had been pooled at a 1:1 ratio. This ensures that there is no well-to-well variability in the relative quantities of RNA from the two spike-in sets. The variance of the log-ratio across these premixed-addition wells provides a baseline level of variability in the protocol (e.g., due to sequencing noise). The variance of volume addition was then estimated as the difference in the variance estimates from the premixed-addition wells and from the wells with separate addition of spike-ins.

We stress that the use of two different spike-in sets in each well is critical to this experiment. Any well-specific biases should cancel out when the log-ratio is computed between sets in the same well. This allows the contribution of the variance of volume addition to be quantified separately from other factors such as the variability of capture efficiency and sequencing depth across wells.

We performed both the premixed and separate-addition experiments on the same plate to avoid plate effects (Hicks et al. 2015; Tung et al. 2017). For the separate-addition experiment, we also reversed the order of addition of the two spike-in sets to determine if this affected the variance estimate. Finally, we generated data from replicate plates to ensure our results were reproducible. This was done in a range of conditions, i.e., using different cell types, by different operators, and with sequencing at different locations.

We used a protocol based on microtiter plates rather than microfluidics as it is easier to customize the spike-in addition step in the former. Our experimental design requires two separate additions of spike-in RNA to each reaction (Methods). This is not straightforward to achieve on, say, the Fluidigm C1 chip, where the added volume for each reagent depends on the design on the reaction chamber. Our focus on data from plate-based protocols reflects their widespread use in single-cell studies (Islam et al. 2011; Wilson et al. 2015; Scialdone et al. 2016; Segerstolpe et al. 2016). Obviously, the procedure we describe here can be adapted to any protocol where the spike-in addition can be easily modified, e.g., plate-based CEL-seq (Hashimshony et al. 2016) or STRT-seq (Islam et al. 2011).

### Mathematical framework for variance decomposition

Denote the log_2_-transformed total read count for well *i* and spike-in set *s* as

$$T_{is}=\log_{2}\left[L_{i}l_{s}V_{is}R_{is}\underset{t_{s}}{\sum }r_{t_{s}}c_{t_{s}}\right]+\varepsilon_{is},$$

where the sum is taken over all unique transcripts *t*_*s*_ in *s*. The other terms are defined as follows:

- $c_{t_{s}}$, a constant specifying the concentration (in terms of transcripts per unit of volume) of *t*_*s*_;
- $r_{t_{s}}$, a constant specifying the optimal transcript molecule-to-cDNA fragment capture rate for *t*_*s*_;
- *R*_*is*_, a random variable representing the average capture efficiency in *i* for all transcripts in *s*;
- *V*_*is*_, a random variable representing the volume of solution of *s* added to *i*;
- *L*_*i*_, a random variable representing the baseline cDNA fragment-to-read conversion rate for *i*; and
- *l*_*s*_, a constant that scales *L*_*i*_ depending on the “sequenceability” of transcripts in *s*.

The product of all of these terms defines the expected number of reads for each *t*_*s*_ in well *i*, and the sum of the products across all *t*_*s*_ is the expected total count of set *s* in *i*. In addition, ɛ_*is*_ represents the effect of sequencing noise on the log-total count, where *E*(ɛ_*is*_) = 0 and $\text{var(}\varepsilon_{is})=\sigma_{lib(s)}^{2}$.

We assume that *R*_*is*_, *V*_*is*_, and ɛ_*is*_ are mutually independent of each other, as they describe separate steps in the protocol. We also assume that *V*_*i*1_ and *V*_*i*2_ are independent for sets *s* = 1 and 2, as each spike-in set is added separately to each well. Similarly, ɛ_*i*1_ and ɛ_*i*2_ are assumed to be independent, as sequencing noise for each transcript should be unaffected by that of other transcripts. (However, *R*_*i*1_ and *R*_*i*2_ are not independent due to well-specific factors affecting capture efficiency for all transcripts.) Further details on these variables are provided in Section 1 of the Supplemental Material.

Let *s* = 1 represent the ERCC spike-in set and *s* = 2 represent the SIRV spike-in set. The log_2_-total count across all spike-in transcripts in the ERCC and SIRV set is *T*_*i*1_ and *T*_*i*2_, respectively. In the experiment where each spike-in set is added separately to each well, we denote the log_2_-ratio of the total counts between the two sets as *θ*_*i*_ = *T*_*i*1_ − *T*_*i*2_ for well *i*. This can also be written as

$$\theta_{i}=\log_{2}(V_{i1})+\varepsilon_{i1}-\log_{2}(V_{i2})-\varepsilon_{i2}+F_{i}+\log_{2}\left[\frac{l_{1}\sum_{t_{1}}r_{t_{1}}c_{t_{1}}}{l_{2}\sum_{t_{2}}r_{t_{2}}c_{t_{2}}}\right],$$

where *F*_*i*_ = log_2_(*R*_*i*1_/*R*_*i*2_) and represents the log-fold change in the average capture efficiency between the two sets (i.e., the difference in behavior of the transcripts). Computing the variance of θ_*i*_ yields

$$\text{var(}\theta_{i})=2\sigma_{vol}^{2}+\sigma_{lib(1)}^{2}+\sigma_{lib(2)}^{2}+\text{var(}F_{i}),$$

where $\sigma_{vol}^{2}$ is the variance of both log_2_(*V*_*i*1_) and log_2_(*V*_*i*2_). The volume addition procedure is the same for each spike-in set, so *V*_*i*1_ and *V*_*i*2_ should have the same distribution. We consider the variance of *F*_*i*_ because *R*_*i*1_ and *R*_*i*2_ are not independent (due to well-specific factors, as previously mentioned).

In the experiment where the spike-in sets are premixed before addition, *V*_*i*1_ = *aV*_*i*2_ for some constant *a* representing the proportions in which the two sets are mixed. (This should be close to unity.) If the same premixed solution is added to each well, the relative volume of ERCC spike-ins to SIRV spike-ins must be constant for all wells. This means that the log_2_-ratio for the premixed experiment is

$$\theta_{i}^{*}=\log_{2}(a)+\varepsilon_{i1}-\varepsilon_{i2}+F_{i}+\log_{2}\left[\frac{l_{1}\sum_{t_{1}}r_{t_{1}}c_{t_{1}}}{l_{2}\sum_{t_{2}}r_{t_{2}}c_{t_{2}}}\right].$$

As *a* is constant for all *i*, the variance of $\theta_{i}^{*}$ becomes

$$\text{var(}\theta_{i}^{*})=\sigma_{lib(1)}^{2}+\sigma_{lib(2)}^{2}+\text{var(}F_{i}).$$

This represents the technical variance attributable to the rest of the scRNA-seq protocol. To obtain an estimate of the variance of the volume addition step, simple arithmetic yields

$$\sigma_{vol}^{2}=\frac{\mathrm{var}(\theta_{i})-\mathrm{var}(\theta_{i}^{*})}{2}.$$

It should be stressed that this variance estimate is relevant to all experiments using the same protocol for spike-in addition, even if the identity or concentration of the spike-in set is different.

Generally, scaling normalization of RNA-seq data is performed by dividing all counts in each library by a library-specific constant, known as the “size factor.” For spike-in normalization, the size factor for cell *i* is directly proportional to the sum of counts for the spike-in transcripts, i.e., $2^{T_{is}}$. This reflects the fact that spike-in normalization aims to eliminate systematic differences in the coverage of spike-in set *s* between cells, thus correcting for well/cell-specific technical biases. (We assume each well contains a cell and will use “cell” and “well” interchangeably in the following text.) Any variance due to volume addition ($\sigma_{vol}^{2}$) or technical noise ($\sigma_{lib(s)}^{2}$) will reduce the precision of *T*_*is*_ and of the size factor estimates (Supplemental Fig. 1), thus reducing the effectiveness of spike-in normalization.

### Estimating the variance of volume addition

Using our mathematical framework, we estimated the variance components based on the data from our mixture experiments. We observed that the log-ratios *θ*_*i*_ and $\theta_{i}^{*}$ computed from each plate were roughly normally distributed (Supplemental Fig. 2). Thus, we fitted a linear model to each set of log-ratios and used the residual variance of the fit as our estimate of var(*θ*_*i*_) or $\text{var(}\theta_{i}^{*})$. Linear models are particularly useful as they allow blocking on additional structure in the experimental design. We used a one-way layout to account for shifts in the mean log-ratio due to addition order or oncogene induction (Methods). The value of *T*_*is*_ was also similar between wells with premixed or separate addition of spike-ins, which simplifies the calculation of $\sigma_{vol}^{2}$ (for details, see Supplemental Fig. 3; Supplemental Material, Section 1). Finally, the order of spike-in addition did not significantly affect the variance estimates for the separate-addition wells in most plates (Supplemental Fig. 4).

**Figure 2.**
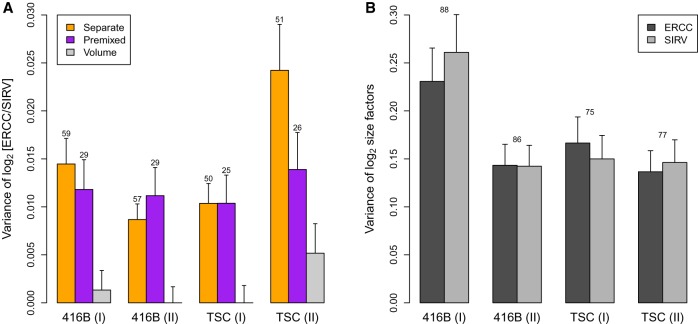
Variance estimates of the log_2_-ratio between the ERCC and SIRV total counts across wells (*A*) or the log_2_-size factors computed from those totals (*B*). For the separate/premixed experiments, each estimate is the residual variance of a linear model fitted to the log-ratios across the corresponding wells on each plate. The variance of volume addition is $\sigma_{vol}^{2}$ in our framework. For the log-size factors, each estimate is the residual variance of a linear model fitted to all cells on each plate. Results are shown for experiments with 416B cells or TSCs, with two replicate plates for each cell type. Error bars represent the standard errors of the estimates, assuming log-values are normally distributed. Numbers represent the residual degrees of freedom used for each estimate—for *B*, this was the same for each spike-in set. Differences between the separate-addition and premixed estimates for each batch were assessed using a one-sided *F*-test, yielding *P*-values of 0.28, 1.00, 1.00, and 0.06 from *left* to *right*.

Our results indicate that $\sigma_{vol}^{2}$ is consistently smaller than $\text{var(}\theta_{i}^{*})$, i.e., the variance in the rest of the protocol (Fig. 2A). Indeed, no significant difference was detected between the estimated var(*θ*_*i*_) and $\text{var(}\theta_{i}^{*})$ of each plate. This indicates that variability of spike-in volume addition is a minor contributor to the technical variability of the spike-in counts. To put these estimates into context, consider that the variance of the log-size factors *T*_*is*_ across cells is at least one order of magnitude larger than $\sigma_{vol}^{2}$ (Fig. 2B). This indicates that the error in the size factors due to variable volume addition is negligible relative to the amount of scaling that is performed to account for differences in sequencing depth and capture efficiency across wells, i.e., var(log_2_*L*_*i*_) and var(log_2_*R*_*is*_). We also computed the variance of the log_2_-ratio of total counts for the mouse genes against one of the spike-in sets. This represents the biological fluctuations in total RNA content across cells and was, again, at least an order of magnitude larger than $\sigma_{vol}^{2}$ (Supplemental Fig. 5). These results show that the variance of volume addition is small compared to other technical and biological sources of variability in a scRNA-seq experiment, and thus is unlikely to have a major effect on spike-in normalization.

### Estimating the variance of differential behavior

The variance of *F*_*i*_ is also relevant as it determines the effect of differences in behavior between distinct sets of transcripts. Even when the average capture efficiency differs between sets, spike-in normalization is still appropriate provided that the fold change in efficiency is the same in all wells. Consider a situation in which there is a consistent increase in efficiency in the spike-in set relative to endogenous transcripts (Svensson et al. 2017). This scales up the counts for the spike-in transcripts in all wells by the same factor, which ultimately cancels out between wells, i.e., the log-fold changes of endogenous or spike-in transcripts between wells are unaffected. However, if the fold change in efficiency varies across wells, the accuracy of spike-in normalization is compromised. This is because specific changes in efficiency for the spike-in transcripts are confounded with general changes in efficiency for all transcripts in the well. Differences in the coverage of spike-in transcripts may not represent technical biases affecting other transcripts, precluding their use for normalizing all counts.

The variance of *F*_*i*_ quantifies the extent to which spike-in normalization is affected by well-to-well differences in efficiency between the spike-in sets. In our mathematical framework, the variance of $\theta_{i}^{*}$ provides an upper bound for the variance of *F*_*i*_. Our estimate of $\text{var(}\theta_{i}^{*})$ is an order of magnitude lower than the estimated variances of the log-size factors in each plate (Fig. 2) and of cellular RNA content (Supplemental Fig. 5). This indicates that the potential variance in the differences in spike-in behavior, although greater than $\sigma_{vol}^{2}$, is still relatively small compared to other biases in the system, e.g., fluctuations in cellular RNA content and well-to-well variability in global capture efficiency. To elaborate, consider that the maximum var(*F*_*i*_) corresponds to an error of $2^{\sqrt{0.015}}\approx 8$% in the size factor estimates. This error is small, especially when we consider that spike-in normalization involves scaling the counts for each cell by at least $2^{\sqrt{0.15}}\approx 30$% in our data sets. These results suggest that variance in spike-in behavior across cells is unlikely to have a strong effect on scaling normalization.

Here, *F*_*i*_ is only computed between two spike-in sets. In practice, the more relevant differences are those between synthetic spike-in and endogenous transcripts. The variance of such differences is likely to be larger than var(*F*_*i*_), given the greater variability in sequence composition and length of endogenous transcripts. Nonetheless, the SIRV and ERCC spike-ins do exhibit some variability in their biophysical properties (Supplemental Fig. 6). For example, the SIRV transcripts have more variable length and lower GC content compared to the ERCC transcripts. This suggests that *F*_*i*_ will include at least some of the differences in behavior between synthetic and endogenous RNA, such that var(*F*_*i*_) can be used as a rough estimate of the magnitude of the associated variability.

### Quantifying the effect of stochastic noise during sequencing

We also performed simulations to gauge the contribution of $\sigma_{lib(s)}^{2}$ to $\text{var(}\theta_{i}^{*})$ (Supplemental Material, Section 2.1). Counts for spike-in transcripts were simulated such that any variability in the log-ratios was only caused by stochastic sampling noise, i.e., $\sigma_{lib(1)}^{2}+\sigma_{lib(2)}^{2}$. Our results suggest that much of the estimated variance of $\theta_{i}^{*}$ in Figure 2 is driven by sampling noise (Supplemental Fig. 7). Specifically, we estimated the variance due to sampling noise to be 0.005–0.012 (using the original spike-in coverage for each plate), compared to estimates of 0.010–0.015 for $\text{var(}\theta_{i}^{*})$ in Figure 2. Both $\sigma_{lib(1)}^{2}+\sigma_{lib(2)}^{2}$ and var(*F*_*i*_) contribute to $\text{var(}\theta_{i}^{*})$, so these results suggest that the contribution of sampling noise is comparable to or greater than the impact of differences in spike-in behavior.

We also observed that the variance due to sampling noise was robust to moderate decreases in the coverage of the spike-in transcripts in this simulation. In ideal experiments, spike-in transcripts would take up 5%–10% of the library size for each cell (50,000–100,000 reads in our data). Upon decreasing coverage in silico, we observed an increase in $\sigma_{lib(1)}^{2}+\sigma_{lib(2)}^{2}$ due to the elevated effect of noise at low counts (Supplemental Fig. 7). However, even at 40%–50% coverage, the variance due to noise was still an order of magnitude lower than the variance due to cell-specific biases (Fig. 2B) or biological variability (Supplemental Fig. 5). These results suggest that spike-in normalization is still reliable when lower amounts of spike-in RNA are added. This is especially relevant to data sets in which the spike-in coverage is lower than recommended, due to difficulties in determining the appropriate concentration of spike-ins to add to each cell when the quantity of endogenous RNA is unknown.

Finally, we performed simulations to assess the effect of noise on the precision of the spike-in size factors themselves (Supplemental Material, Section 2.2). This was performed using our 416B and TSC data sets as well as public data from existing studies (Grün et al. 2014; Islam et al. 2014; Buettner et al. 2015; Kolodziejczyk et al. 2015; Scialdone et al. 2015; Zeisel et al. 2015; Hashimshony et al. 2016). In each data set, we observed that sampling noise resulted in ≈5% error in the estimates for the spike-in size factors (Supplemental Fig. 8). In comparison, the size factors routinely varied by >30% across cells (Supplemental Fig. 9). Thus, the loss of precision due to noise is small and can probably be ignored during spike-in normalization.

### Assessing the downstream effect of variability with simulations

We assessed whether the results of downstream analyses using spike-in normalization were sensitive to fluctuations in the total spike-in counts due to variability in spike-in addition, behavior, or sequencing noise. First, we obtained data from scRNA-seq experiments that used spike-in RNA. This included a number of public data sets (Grün et al. 2014; Islam et al. 2014; Buettner et al. 2015; Kolodziejczyk et al. 2015; Scialdone et al. 2015; Segerstolpe et al. 2016) as well as our 416B and TSC data. We then performed analyses such as detection of differentially expressed genes (DEGs) and highly variable genes (HVGs), as well as dimensionality reduction and clustering of cells. This was done without any modification of the data to obtain a set of “original results.”

Next, we designed simulations based on each of the real data sets (Methods). Briefly, the total spike-in count for each well was rescaled by a randomly sampled factor with variance equal to our experimental estimate of spike-in variance. Counts for the individual spike-in transcripts were rescaled to reflect this new total, thus yielding a simulated data set. Downstream analyses were performed using the original counts for the endogenous genes and the simulated counts for the spike-in transcripts. The new results were then compared to the original set of results from each analysis. Any differences indicate that the analysis is sensitive to spike-in variability in real experiments. The advantage of this simulation design is that only the spike-in counts are modified. No simulations or resampling were performed for the counts of the endogenous genes, preserving the realistic nature of the data in each simulation and ensuring that only spike-in variability can cause differences in the analysis results.

**Figure 3.**
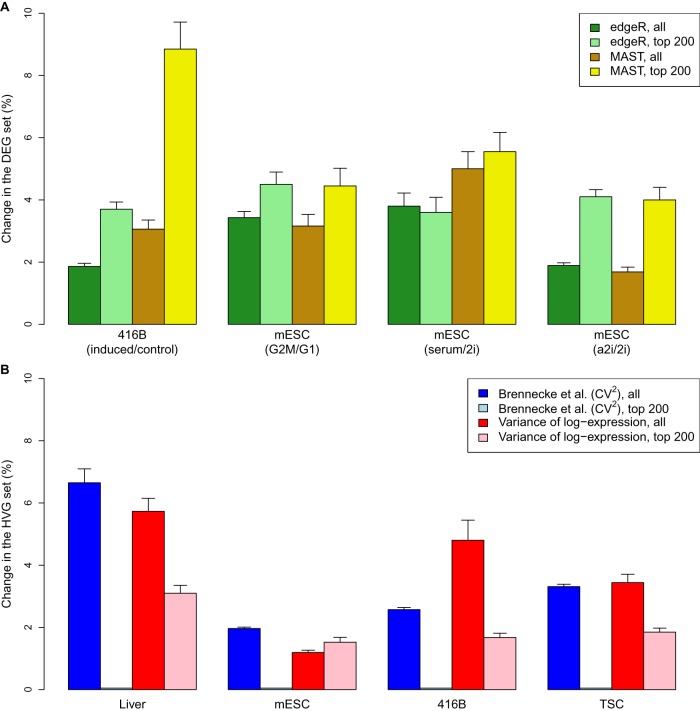
Effect of spike-in variability on DEG or HVG detection in simulated data. (*A*) The percentage change in the set of DEGs detected in each data set at a FDR of 5% by edgeR or MAST. This was also calculated for the top set of 200 DEGs with the smallest *P*-values. Simulations were performed to detect DEGs in our 416B data set after inducing expression of a *CBFB-MYH11* oncogene compared to an mCherry control (Methods), between mouse embryonic stem cells (mESCs) in G1 and G2/M phases of the cell cycle (Buettner et al. 2015), or between mESCs cultured in different conditions—serum, ground state (2i), or alternative ground state (a2i) (Grün et al. 2014; Kolodziejczyk et al. 2015). (*B*) The percentage change in the set of HVGs detected in each data set at a FDR of 5%, using the method of Brennecke et al. (2013) based on the squared coefficient of variation (CV^2^) or with a method based on the variance of log-expression. This was also calculated for the top set of 200 HVGs with the smallest *P*-values. Simulations were performed to detect HVGs in our 416B and TSC data sets, in liver cells (Scialdone et al. 2015), and in mESCs (Kolodziejczyk et al. 2015). All values represent the mean of 20 simulation iterations, and error bars represent standard errors.

For DEG detection, we applied edgeR (Robinson et al. 2010) and MAST (Finak et al. 2015) to the original and simulated data after spike-in normalization. edgeR represents methods designed for DE analyses of bulk RNA-seq data, whereas MAST represents bespoke single-cell methods. In both cases, we observed only minor (<5%) changes to the set of significant DEGs upon introducing spike-in variability in each data set (Fig. 3A). Similar results were also observed in the top 200 DEGs with the smallest *P*-values, with <10% of the genes in the set changing across iterations in all scenarios. For HVG detection, we used methods based on the coefficient of variation (Brennecke et al. 2013) or the variance of log-expression values (Lun et al. 2016b). Again, only minor changes were observed in most data sets (Fig. 3B), for both the set of significant HVGs and for the top 200 HVGs with the smallest *P*-values. These results suggest that the detection and ranking of DEGs and HVGs are largely robust to variability in spike-in volume or behavior. Indeed, genes that were not consistently detected across simulation iterations tended to have weak log-fold changes for DEGs or small biological components for HVGs (Supplemental Fig. 10). This is expected because genes on the borderline of significance are more susceptible to random fluctuations in the size factors.

**Figure 4.**
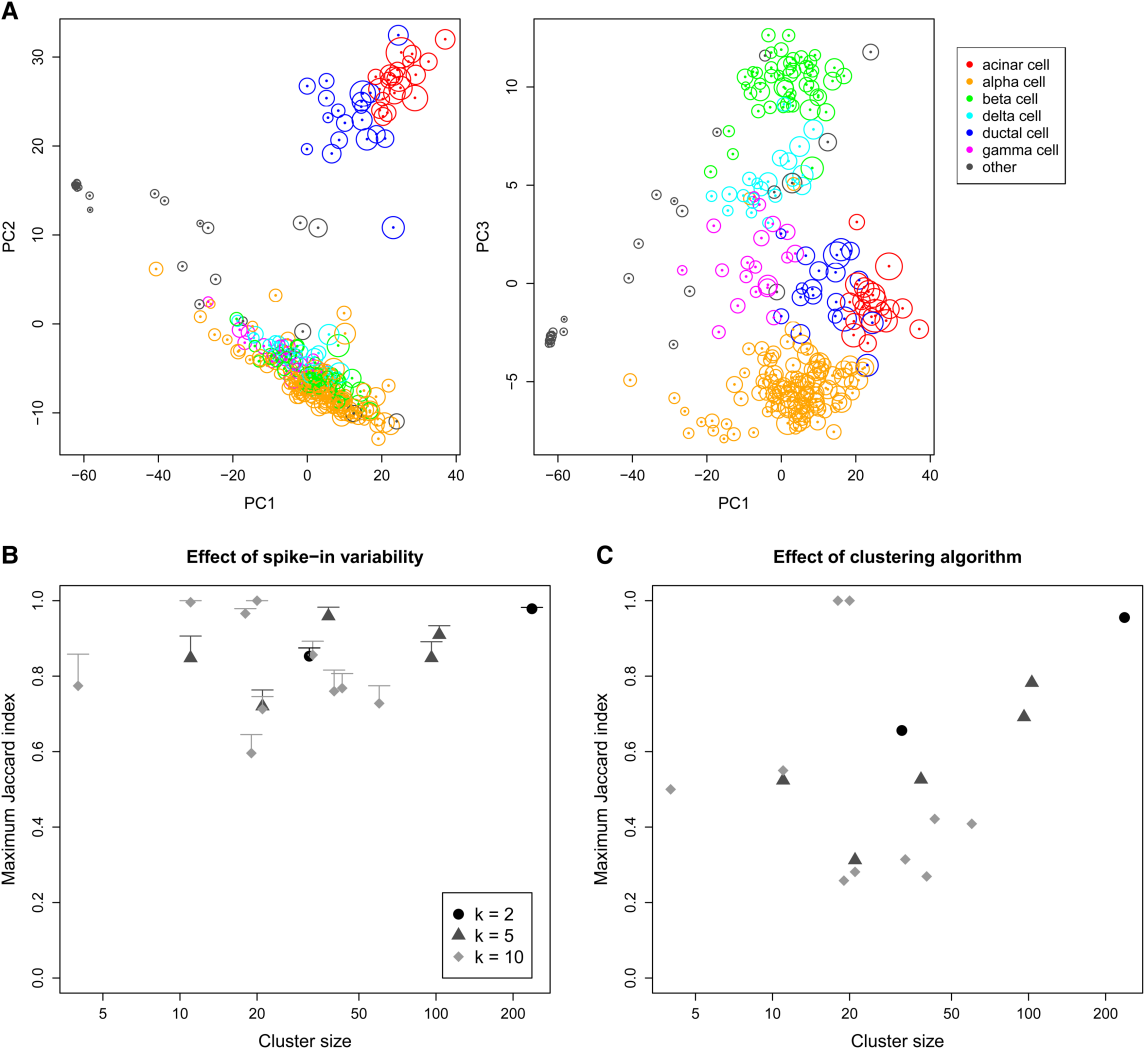
Effect of spike-in variability on dimensionality reduction and clustering in simulated data, based on real scRNA-seq data for cells extracted from a healthy human pancreas (Segerstolpe et al. 2016). (*A*) PCA plots of the first three principal components, in which each cell is colored according to its annotated cell type from the original study. The circle *around* each cell contains 95% of remapped locations across the simulation iterations, and represents the deviation in location due to spike-in variability. (*B*) Clusters were identified from the original data by hierarchical clustering with Ward's criterion, followed by a tree cut with *k* of 2, 5, or 10. This was repeated at each simulation iteration, and the maximum Jaccard index between each original cluster and any of the simulated clusters at the same *k* was computed. Each value represents the mean of 20 simulation iterations, and the error bars represent standard errors. (*C*) The maximum Jaccard index for each original cluster generated with Ward's criterion compared to clusters generated from complete-linkage clustering of the original data.

For dimensionality reduction, we restricted ourselves to principal components analysis (PCA) on the normalized expression profiles of all cells. Although *t*-distributed stochastic neighbor embedding (van der Maaten and Hinton 2008) is commonly used, its robustness is difficult to evaluate due to its randomness. We used a scRNA-seq study of the human pancreas (Segerstolpe et al. 2016) to generate PCA plots of the first three principal components for both the original and simulated data. At each simulation iteration, coordinates of all cells in the simulated plots were mapped onto the corresponding original plots to determine the sensitivity of the original locations to spike-in variability. Figure 4A indicates that changes in the location of each cell across simulation iterations were generally minor. In particular, movement of cells across iterations did not compromise the separation of different cell types. Thus, spike-in variability does not appear to affect the visual interpretation of PCA plots.

Finally, we performed hierarchical clustering and applied a tree cut to identify clusters of cells in the original data. This was repeated at each simulation iteration to obtain a corresponding set of simulated clusters. For each original cluster, we computed the Jaccard index with respect to each of the simulated clusters and recorded the maximum value across all simulated clusters. A large maximum Jaccard index means that most of the cells in the original cluster are still grouped together in the simulation, i.e., the original cluster is (mostly) successfully recovered in one of the simulated clusters. We observed that the maximum Jaccard indices were moderate to large (Fig. 4B), with values above 0.6 for most of the original clusters. To put this into context, we reclustered the original data using a different algorithm. This yielded smaller Jaccard indices for all clusters (Fig. 4C), indicating that spike-in variability has less effect on the results than the choice of clustering method.

## Discussion

In this article, we performed mixture experiments to quantify the variability of spike-in RNA addition across wells in a plate-based scRNA-seq protocol. We also obtained a rough estimate of the well-to-well variability in the differences in behavior between two different sets of spike-in transcripts. Both values were at least an order of magnitude smaller than the variance of spike-in coverage across cells, suggesting that differences in spike-in volume or behavior were not major sources of error in the context of spike-in normalization. This was supported by simulations in which the introduction of realistic levels of spike-in variance yielded only minor changes in the results of DEG and HVG analyses as well as PCA and clustering. Our results indicate that spike-in normalization is reliable enough for routine use in scRNA-seq data analyses. The common criticisms of using spike-in RNA for scaling normalization are only weakly relevant, if at all, to single-cell transcriptome studies, and can generally be ignored.

Our conclusions differ from those of Risso et al. (2014), in which spike-in normalization is not considered reliable enough for analyses of bulk RNA-seq data. We speculate that this difference may be due to the difficulty of adding an appropriate amount of spike-in RNA at the population level. For example, should spike-in RNA be added at a constant ratio with respect to the concentration of endogenous RNA, or to the number of cells in the sample? If the endogenous RNA concentration or the number of cells determines the amount of spike-in RNA to be added, these will need to be experimentally quantified for each sample. In that case, how accurate is the quantification, and what effect do errors have on the downstream analysis? These questions are not relevant to single-cell experiments for which the obvious approach is to add the same amount of spike-in RNA to each individual cell.

It is worth mentioning another common criticism of the use of spike-in RNA—namely, that the optimal concentration (to reach the suggested 5%–10% of total library size) depends on the amount of endogenous RNA in each cell. This is not straightforward to gauge for biological systems that are not well studied. If insufficient spike-ins are added, sampling noise will increase and the precision of spike-in normalization will deteriorate. However, we do not consider this to be a matter of reliability. It is not surprising that suboptimal performance is obtained when inappropriate concentrations of reagents are used, and spike-in RNA is no exception. Pilot experiments can be performed to identify the most suitable spike-in concentration to use for a given biological system, just like they would be used to determine the optimal dissociation conditions, lysis buffer, amplification cycles, and so on. Once the optimal concentration is determined, it can be used for all experiments on that system, and the choice of concentration ceases to be an issue. In contrast, the variability of volume addition and of spike-in behavior cannot be easily controlled even if all other parameters are optimized.

We used the Smart-seq2 protocol in our study to reflect its widespread use in the scRNA-seq literature. However, our estimate of $\sigma_{vol}^{2}$ is agnostic to how reverse transcription, amplification, and sequencing were performed, as these steps are represented by other mathematical terms. Thus, we expect our conclusions to be broadly applicable to any scRNA-seq protocol where spike-in RNA is added in a similar manner (using repeater pipettes) (Methods). Different results will be obtained using other methods for spike-in addition, e.g., with robotics systems or microfluidics, in which volume handling may be even more precise. Our experimental framework may also be useful for evaluating the precision of spike-in addition when developing new scRNA-seq protocols or setting up existing protocols in new laboratories, to ensure that spike-in RNA is added correctly to each cell.

The term var(*F*_*i*_) represents the variability in the difference in behavior between the SIRV and ERCC spike-in sets across wells. However, arguably a more relevant quantity is the variability in the difference *P*_*is*_ between synthetic spike-in and endogenous RNA, as this affects the accuracy of normalization. It may be possible to obtain a rough estimate of var(*P*_*is*_) by using pooled cellular RNA from another organism as one of the spike-in sets (Brennecke et al. 2013), so that $\text{var(}\theta_{i}^{*})$ provides an upper bound on the variance in the differences in behavior between synthetic and endogenous RNA. We chose not to do so because of the difficulty in reproducibly using the same pool of cellular RNA across batches and in calibrating the concentration of RNA to be added to each well. Use of UMI counts may also provide a tighter bound on var(*F*_*i*_) or var(*P*_*is*_) by reducing the contribution of amplification noise to $\sigma_{lib(s)}^{2}$ and $\text{var(}\theta_{i}^{*})$. Another consideration with endogenous RNA is the variability of lysis between cells, which we neglect to consider in our framework; this is inherently difficult to assess with external spike-in RNA and may require other methods to quantify.

One interesting question is how to choose between spike-in normalization and approaches that assume a non-DE majority of genes. This choice depends on whether total RNA content in each cell is of interest (Lun et al. 2016b). Spike-in normalization will preserve changes in total RNA content between cells, whereas non-DE methods will treat such changes as bias (as a majority of genes are affected) and remove them. This suggests that spike-in normalization is preferable in applications in which changes in total RNA content can be easily associated with a biological process, e.g., T cell activation and cell cycling. In contrast, non-DE normalization may be more suitable for comparisons between distinct cell types, where the up- or down-regulation of specific genes (conditional on the total RNA content of the cell) is more informative. Obviously, this choice is subject to the experimental context. If a non-DE majority cannot be assumed, spike-in normalization should be used; conversely, spike-in RNA cannot be easily added in droplet-based techniques, thus requiring non-DE methods.

We stress that our study only examined the reliability of spike-ins for “relative” normalization, i.e., to make counts comparable across cells. We do not consider the reliability of spike-ins for absolute quantification, i.e., to determine the number of molecules of each transcript in each cell. This is more difficult to evaluate as accuracy is affected by the magnitude of the differences in the behavior of spike-in and endogenous transcripts. In contrast, relative normalization is only affected by variability in the differences in behavior across wells, as we have previously discussed.

## Methods

### Obtaining and culturing 416B cells and TSCs

The murine multipotent myeloid progenitor cell line 416B (Dexter et al. 1979) was stably transduced with a TetOn construct of the *CBFB-MYH11* (CM) oncogene (type A cDNA), using an in-frame F2A-mCherry protein as a reporter. As a control, cells were alternatively transduced with a version of the construct lacking the CM cDNA. Cells were maintained in RPMI medium, supplemented with 10% fetal calf serum and antibiotics. Expression of the CM oncogene or the mCherry control was induced by treatment with 1 µg/mL of doxycycline, and induction was confirmed after 24 h by measurement of mCherry levels by fluorescence activated cell sorting (BD Fortessa).

Murine TSCs were kindly provided by Dr. Jennifer Nichols (Wellcome Trust and MRC Cambridge Stem Cell Institute) and cultured by Liliana Antunes (Wellcome Trust Sanger Institute) on mouse embryonic fibroblast (MEF) feeders with TSC culturing medium (a combination of 70% MEF conditioned media [R&D systems] and 30% RPMI 1640, supplemented with 20% FBS, 2 mM L-glutamine, 1 mM sodium pyruvate, 100 µM β-mercaptoethanol, 25 ng/mL human recombinant FGF4 [R&D systems], and 1 µg/mL heparin [Tocris Bioscience]). To prepare for single-cell sorting, cells were harvested with trypsin, and MEF feeders were depleted by plating the cells onto a gelatinized plate followed by incubation for 1 h at 37°C on TSC culturing medium. The supernatant containing TSCs was used for sorting.

### Spike-in mixture experiments with Smart-seq2

Single-cell RNA sequencing was performed using an adaptation of the previously described Smart-seq2 protocol (Picelli et al. 2014). Single 416B cells or TSCs were sorted into individual wells of a 96-well microtiter plate. Each well contained 2.3 µL of lysis buffer with RNase inhibitor (Ambion) in a 0.2% (v/v) Triton X-100 solution. Reverse transcription (RT) was performed in a final volume of 13.2 µL per well, containing 1 µM of oligo-dT (Sigma-Aldrich), 1.04 mM of each dNTP (Thermo Fisher), 100 units of SuperScript II retrotranscriptase (Invitrogen/Thermo Fisher), 5 units of RNase inhibitor (Ambion), 5 mM of DTT, 1 M of Betaine (Sigma-Alrich), 6 mM of MgCl_2_ (Ambion), and 1 µM of TSO primer (Exiqon). Preamplification was performed in a total volume of 27 µL that contained 13.5 µL of HiFi Hotstart ReadyMix (2×; KAPA Biosystems) and 0.1 µM of IS PCR primer (Sigma-Aldrich). After 23 cycles of amplification, samples were cleaned with 80% (v/v) of Ampure beads (Beckman Coulter). Sequencing libraries were prepared using the Nextera XT DNA sample preparation kit (Illumina). This was repeated to obtain several batches of sequencing data, with each batch consisting of one plate of cells of the same type.

To perform the mixture experiments, spike-in RNA was mixed into the RT reagent solution and added to each well. This was done such that each well contained 0.1 µL of a 1:3,000,000 dilution of the ERCC RNA Spike-In Mix (Invitrogen/Thermo Fisher) and 0.12 µL of a 1:3,000,000 dilution of the Spike-in RNA Variant (SIRV) Control Mix E0 (Lexogen). Two separate solutions of RT reagents were prepared for the different spike-in sets. For one-third of the wells, addition of the two spike-in sets was performed separately with the RT + ERCC solution first and the RT + SIRV solution second. For another one-third of the wells, the order was reversed, i.e., with the RT + SIRV solution first and the RT + ERCC solution second. For the remaining wells, the RT + SIRV and RT + ERCC solutions were premixed in a 1:1 ratio, and the RT + SIRV + ERCC mixture was added twice to each well. Each addition was performed independently for each well, using a repeater pipette dispensing 2 µL at a time.

Sequencing of the 416B libraries was performed by the Genomics Core facility at the Cancer Research UK Cambridge Institute. The first batch of libraries was sequenced on an Illumina HiSeq 2500 machine generating 125-bp single-end reads, whereas the second batch was sequenced on an Illumina HiSeq 4000 machine generating 50-bp single-end reads. Sequencing of the TSC libraries was performed at the Wellcome Trust Sanger Institute after library preparation by the Single Cell Genomics Core facility. Both batches were sequenced on an Illumina HiSeq 4000 machine generating 75-bp paired-end reads.

### Data analysis for the mixture experiments

Reads were mapped to the mm10 build of the mouse genome, including sequences of transcripts in the ERCC (https://tools.thermofisher.com/content/sfs/manuals/ERCC92.zip) and SIRV (https://www.lexogen.com/wp-content/uploads/2015/11/SIRV_Sequences 151124.zip) spike-in sets. (The sequence of the *CBFB-MYH11* oncogene was also included in the reference when aligning data from 416B cells.) Mapping was performed using the subread aligner v1.5.1 (Liao et al. 2013) in RNA-seq mode with unique alignment. The 416B data were aligned in single-end mode, whereas the TSC data were aligned in paired-end mode. Reads with mapping qualities greater than or equal to 10 were assigned to exonic regions of genes using the featureCounts function in the Rsubread package v1.24.1 (Liao et al. 2014). Genes were defined using Ensembl v82 annotation for the GRCm38 mouse assembly and annotation for the ERCC and SIRV transcripts. This yielded a count for each endogenous gene and spike-in transcript in each well. Mapping and counting statistics for each batch of libraries are summarized in Supplemental Table 1.

To evaluate spike-in quality, we verified that the total spike-in count (ERCC + SIRV) in each well comprised 5%–10% of the total library size (Supplemental Fig. 3). This corresponds to the amount of spike-in RNA that we aimed to add to each well and was consistent across wells within each plate. The coverage of each ERCC transcript was directly proportional to its theoretical concentration in the spike-in mixture (Supplemental Fig. 11), and the distribution of average read counts across spike-in transcripts or endogenous genes was consistent across plates (Supplemental Fig. 12). These diagnostics indicate that the spike-in transcripts were successfully captured, sequenced, and processed into counts for most wells. We removed any wells where the log-total count for either spike-in set or for the endogenous genes was more than three median absolute deviations below the median value for each plate. It is likely that capture or sequencing failed for these wells, so they were not used for variance estimation. In addition, we examined the effect of index switching (Sinha et al. 2017) in each data set generated on the HiSeq 4000 and found it to be negligible (Supplemental Material, Section 3; Supplemental Fig. 13).

Variance components were estimated from the libraries generated from a single plate. In each well, the sum of counts across all transcripts in each spike-in set was computed, and the log_2_-ratio between the ERCC and SIRV sums was calculated. To estimate var(*θ*_*i*_), a linear model with a one-way layout was fitted to the log-ratios for all wells where the two spike-in sets were added separately. In each plate of the 416B data set, each combination of treatment (control or oncogene-induced) and spike-in addition order (ERCC or SIRV first) was treated as a group in the one-way layout. In each plate of the TSC data, only the spike-in addition order was used to define the groups. After fitting the model, the mean of the squared residual effects was used as an estimate of var(*θ*_*i*_). This was repeated for $\text{var(}\theta_{i}^{*})$ using all wells where premixed spike-ins were added. Here, addition order was irrelevant, so the one-way layout contained only the two treatment groups in the 416B data set. Similarly, only a single group was defined for the TSC data. Linear modeling ensures that any changes in the mean log-ratio across groups do not inflate the variance estimate. Note that we fit linear models to each plate separately, to check whether the estimates are consistent across replicate plates.

To detect differences in the variance estimates for premixed and separate addition, an *F*-test for the equality of variances was applied. Under the null hypothesis of equal variances computed from independent data, the ratio of the variances $\sigma_{1}^{2}/\sigma_{2}^{2}$ should follow an *F*-distribution on *n*_1_ and *n*_2_ degrees of freedom, where *n*_1_ and *n*_2_ are the residual degrees of freedom used to estimate $\sigma_{1}^{2}$ and $\sigma_{2}^{2}$, respectively. This can either be one-sided (i.e., $\sigma_{1}^{2}\leq \sigma_{2}^{2}$ under the null), in which case the upper tail probability at the observed ratio is taken as the *P*-value; or it can be two-sided, in which case the *P*-value is defined as twice the smaller of the two tail probabilities. Significant differences were defined by rejecting the null hypothesis at a type I error rate of 5%. We calculated $\sigma_{vol}^{2}$ from estimates of var(*θ*_*i*_) and $\text{var(}\theta_{i}^{*})$, using the expression described above. However, if the difference between var(*θ*_*i*_) and $\text{var(}\theta_{i}^{*})$ was negative, $\sigma_{vol}^{2}$ was set to zero instead. To assess the effect of the order of spike-in addition, a linear model was fitted to the subset of relevant wells on each plate to obtain an order-specific variance estimate.

### Simulation design for resampling spike-in variability

For each data set, we compute *T*_*is*_ for each cell *i* and spike-in set *s*. To simplify the design of the simulations, we only consider the ERCC spike-in set here, i.e., *s* = 1. The variance of *T*_*is*_ is

$$\text{var(}T_{is})\approx \sigma_{lib(s)}^{2}+\sigma_{vol}^{2}+\text{var(}\log_{2}R_{is})+\text{var(}\log_{2}L_{i}),$$

where the approximation assumes that *L*_*i*_ is independent of the other random variables that contribute to *T*_*is*_ (for more detail, see Supplemental Material, Section 1). Let *R*_*is*_ = *R*_*i*0_*P*_*is*_, where *R*_*i*0_ is a random variable representing the well-specific average capture efficiency of endogenous transcripts, and *P*_*is*_ is the fold change in average efficiency of the transcripts in *s* over their endogenous counterparts. We assume that *R*_*i*0_ and *P*_*is*_ are independent for each well, and that var(log_2_*P*_*is*_) can be approximated with var(*F*_*i*_), i.e., the well-to-well variability in relative capture efficiency between the two spike-in sets is similar to that between spike-ins and endogenous transcripts. This means that

$$\text{var(}T_{is})\approx \sigma_{lib(s)}^{2}+\sigma_{vol}^{2}+\text{var(}F_{i})+\text{var(}\log_{2}R_{i0})+\text{var(}\log_{2}L_{i}),$$

i.e., the variance of *T*_*is*_ is a sum of the variances of its component terms. The above approximation allows us to account for the measured $\sigma_{vol}^{2}$, $\sigma_{lib(s)}^{2}$, and var(*F*_*i*_) when simulating new values for *T*_*is*_.

Let us denote $x^{2}=\sigma_{vol}^{2}+\text{var(}F_{i})+\sigma_{lib(s)}^{2}$, representing the total variance in the log_2_-total count of one spike-in set *s* due to variable addition, capture efficiency, and sequencing noise. We use the estimated $\text{var(}\theta_{i}^{*})\approx 0.015$ in Figure 2A as our estimate ${\hat{x}}^{2}$ of the upper bound of *x*^2^. This is based on the fact that $\sigma_{vol}^{2}$ is near-zero in Figure 2A, whereas $\text{var(}\theta_{i}^{*})=\sigma_{lib(1)}^{2}+\sigma_{lib(2)}^{2}+\text{var(}F_{i})$ and thus provides an upper bound on $\mathrm{var}(F_{i})+\sigma_{lib\left(s\right)}^{2}$for any *s*. We also denote ${\hat{\sigma }}_{s}^{2}$ as the estimate of var(*T*_*is*_) across wells and ${\hat{\mu }}_{s}$ as the estimate of *E*(*T*_*is*_). For each well *i*, we compute a simulated log_2_-total $T_{is}^{*}$ as

$$T_{is}^{*}=(T_{is}-{\hat{\mu }}_{s})\sqrt{1-\frac{{\hat{x}}^{2}}{{\hat{\sigma }}_{s}^{2}}}+{\hat{\mu }}_{s}+X_{i},$$

where $X_{i}∼\text{Normal(}0,{\hat{x}}^{2})$ and is independently sampled for each well. This approach ensures that $\text{var(}T_{is}^{*})={\hat{\sigma }}_{s}^{2}$. In contrast, if *X*_*i*_ were directly added to *T*_*is*_, the variance of $T_{is}^{*}$ would be inflated as *x*^2^ is already present in var(*T*_*is*_), i.e., the contribution of spike-in variance would be doubled.

Counts for the library generated from each well were rescaled to reflect the new, simulated log-total. A quantile adjustment approach was used to preserve the empirical mean–variance relationship. Briefly, a negative binomial generalized linear model (NB GLM) was fitted to the counts across all wells for each spike-in transcript, using the glmFit function in edgeR (Robinson et al. 2010; McCarthy et al. 2012) with a design matrix containing all experimental factors in the current data set. The value of *T*_*is*_/log_2_(*e*) was used as the offset for well *i* during GLM fitting. The NB dispersion was also estimated for each transcript using the estimateDisp function without empirical Bayes shrinkage. For each transcript *t*, we assumed that the count *y*_*ti*_ for well *i* was sampled from a NB distribution with mean equal to the corresponding fitted value of the GLM and dispersion equal to the estimated transcript-specific value. We scaled the NB mean by $2^{T_{is}^{*}-T_{is}}$ to obtain a modified NB distribution. Using the q2qnbinom function (Robinson and Smyth 2008), we calculated the lower tail probability of *y*_*ti*_ in the original distribution and identified the corresponding quantile with the same tail probability in the modified distribution. This new quantile was used as the simulated count for transcript *t* in *i*.

### Evaluating the robustness of DEG detection

We used a number of data sets to test the effect of spike-in variability on DEG detection. This included our 416B data, in which DEGs were detected between control and oncogene-induced cells; and public data sets involving mESCs, in which DEGs were detected between G1 and G2/M phases of the cell cycle (Buettner et al. 2015) or between different culture conditions (Grün et al. 2014; Kolodziejczyk et al. 2015). Access to each public data set is described in Section 4 of the Supplemental Material. In each study, DEGs were detected between conditions using edgeR and MAST. Implementation details of each method are provided in Section 5 of the Supplemental Material. Briefly, normalization was performed by scaling the counts (explicitly or via offsets) such that the spike-in totals were the same between cells. The set of DEGs in the original data was then identified at a FDR of 5%. This procedure was repeated for the simulated data, and the number of genes that were detected in the original results and not in the simulated results (or vice versa) was recorded as a proportion of the total number of original DEGs. The proportion of the top 200 genes with the smallest *P*-values that were different between the original and simulated results was also computed. This was repeated for 20 simulation iterations, and the average proportion across iterations was reported for each method.

### Evaluating the robustness of HVG detection

We used several data sets to test the effect of spike-in variability on HVG detection. This included our 416B and TSC data sets, as well as public data sets involving mESCs (Kolodziejczyk et al. 2015) or liver cells (Scialdone et al. 2015). In each data set, spike-in normalization was performed, and HVGs were detected using two approaches based on spike-in counts. The first approach is based on the method of Brennecke et al. (2013), in which the squared coefficient of variation for each gene is tested for a significant increase above technical noise. The second approach is based on the variance of the log-normalized expression values (Lun et al. 2016b), which provides some more robustness against outlier expression patterns. Each method was applied on the original and simulated data, and a set of significant HVGs was detected at a FDR of 5%. The proportion of HVGs that were detected in the original results and not in the simulated results (or vice versa) was computed. Similarly, we computed the proportion of the top 200 genes with the lowest *P*-values that differed between the original and simulated results. This was repeated for 20 simulation iterations and the average proportion across iterations was reported for each method. See Section 4 of the Supplemental Material for details on public data access and Section 5 for the implementation details of each HVG detection method.

### Evaluating dimensionality reduction and clustering

We obtained count data from a study of pancreatic islet cells (Supplemental Material, Section 4; Segerstolpe et al. 2016). Spike-in normalization was performed and a set of HVGs was defined using the variance-of-log-expression method. PCA plots of the first three components were constructed from the matrix of log-expression values for the HVGs. This process—including HVG detection—was repeated with the simulated data after introducing spike-in variability. To compare each simulated PCA plot to the original plot, the coordinates of each cell in the former were mapped onto the latter by rescaling and rotation. Robustness was assessed based on the spread of remapped coordinates across all simulation iterations for each cell. See Section 5 in the Supplemental Material for details.

To test the robustness of clustering, the matrix of Euclidean distances between cells was computed from the HVG log-expression values. Hierarchical clustering was performed using the Ward criterion, and the resulting dendrogram was cut into 2, 5, or 10 clusters. (This was done using the hclust and cutree commands, respectively, from the stats package.) This process was repeated with the simulated data, and the Jaccard index between every pair of simulated and original clusters was computed. For each original cluster, the maximum Jaccard index across all simulated clusters was recorded at each simulation iteration. This value represents the extent to which the membership of the original cluster was preserved in the most similar simulated cluster. We also compared the original clusters to those generated from complete-linkage clustering of the original HVG log-expression values.

### Software availability

The R code, which was used for the statistical analysis and simulations, is available in the Supplemental Code or at https://github.com/MarioniLab/SpikeIns2016.

## Data access

Data from this study have been submitted to the ArrayExpress database (https://www.ebi.ac.uk/arrayexpress/) under accession number E-MTAB-5522.

## Supplementary Material

Supplemental Material
