# Supplementary material for: Assessing the reliability of spike-in normalization for analyses of single-cell RNA sequencing data
Source: Genome Res. 2017 Nov;27(11):1795–806. doi: 10.1101/gr.222877.117 (PMC5668938; doi:10.1101/gr.222877.117)
Supplement: Supplemental Material [file supp_27_11_1795__index.html]

Assessing the reliability of spike-in normalization for analyses of single-cell RNA sequencing data — Supplemental Material 

# Assessing the reliability of spike-in normalization for analyses of single-cell RNA sequencing data

## Supplemental Material

undefined

- Supplemental\_Materials.pdf
- Supplemental\_Code.zip
