# Supplementary material for: Assessing the reliability of spike-in normalization for analyses of single-cell RNA sequencing data
Source: Genome Res. 2017 Nov;27(11):1795–806. doi: 10.1101/gr.222877.117 (PMC5668938; doi:10.1101/gr.222877.117)
Supplement: Supplemental Material [file supp_gr.222877.117_Supplemental_Materials.pdf]

Assessing the reliability of spike-in normalization for analyses of single-cell RNA sequencing data

Supplemental Materials

by

Aaron T. L. Lun[1], Fernando J. Calero-Nieto[2], Liora Haim-Vilmovsky[3,4], Berthold Göttgens[2], John C. Marioni[1,3,4]

[1]Cancer Research UK Cambridge Institute, University of Cambridge, Li Ka Shing Centre, Robinson Way, Cambridge CB2 0RE, United Kingdom

[2]Wellcome Trust and MRC Cambridge Stem Cell Institute, University of Cambridge, Wellcome Trust/MRC Building, Hills Road, Cambridge CB2 0XY, United Kingdom

[3]EMBL European Bioinformatics Institute, Wellcome Genome Campus, Hinxton, Cambridge CB10 1SD, United Kingdom

[4]Wellcome Trust Sanger Institute, Wellcome Genome Campus, Hinxton, Cambridge CB10 1SA, United Kingdom

October 2, 2017

1 Further interpretation of the mathematical terms

R_{is} was introduced as the average capture efficiency in well i for all transcripts in set s. It ranges from 0 to 1 and scales r_{t_s} to determine the actual capture rate for each t_s. The most obvious interpretation of R_{is} (and r_{t_s}) is that of the efficiency of reverse transcription, but it also describes the efficiency of PCR amplification and tagmentation in Smart-seq2. Thus, no additional variables are necessary for the latter steps.

The term $l_s L_i$ describes the rate at which reads are obtained from cDNA fragments during high-throughput sequencing. The l_s constant represents the average sequencing efficiency for transcripts in s, as well as factors such as mappability that affect the final counts. The interpretation of L_i depends on whether library quantification was performed to equalize the amount of cDNA from each well prior to sequencing. If not, L_i will be constant across wells, with its exact value depending on the sequencing depth. However, if quantification was performed, L_i will theoretically depend on the other variables that contribute to T_{is}. Specifically,

$$L_i \approx D_i \left[\sum_{s\in\{1,2\}} \left(l_s V_{is} R_{is} \sum_{t_s} r_{t_s} c_{t_s} \right) + l_0 R_{i0} \sum_g r_g N_{ig} \right]^{-1}$$

where D_i is a random variable representing the total sequencing depth for well i (in reads); N_{ig} is a random variable specifying the number of transcripts for each endogenous gene g in i; and l_0, R_{i0} and r_g are the equivalents to l_s, R_{is} and r_{t_s} for endogenous genes. In practice, L_i is effectively independent of V_{is} and R_{is} for any particular spike-in set s. This is because the denominator of the above expression is dominated by the vastly larger number of cDNA fragments from the set of endogenous genes. Any correlation between L_i and the other terms would be negligible compared to the biological variance of expression, i.e., $\text{var}(N_{ig})$.

The error term ε_{is} denotes the variability due to sequencing noise in the counts for spike-in set s. We defined its variance as $\sigma^2_{lib(s)}$, which implicitly assumes that there is no relationship between the variance and the mean of T_{is}. This is a strong assumption given that mean-variance relationships are often observed in RNA-seq data (McCarthy et al., 2012; Law et al., 2014). However, we note that the distribution of T_{is} across wells with separate addition of spike-ins is similar to that across wells with premixed addition on the same plate (Supplementary Figure 3). If the mean of T_{is} does not change between separate/premixed experiments, neither will the value of $\sigma^2_{lib(s)}$, regardless of the nature of the mean-variance relationship. This suggests that $\sigma^2_{lib(s)}$ will not change between $\text{var}(\theta_i)$ and $\text{var}(\theta^*_i)$, allowing calculation of σ^2_{vol} from their difference.

2 Computing the contribution of stochastic noise

2.1 Calculating the effect of noise on the log-ratios

We performed simulations to obtain a rough estimate of the contribution of $\sigma^2_{lib(s)}$ to the variance of θ^*_i. For each plate in each data set (416B or TSC), we extracted the spike-in count data for the premixed wells. For each spike-in set (SIRV or ERCC), the log-transformed sum of counts across all transcripts in that set was used as the GLM offset. The NB dispersion was estimated for each spike-in transcript using the estimateDisp function from edgeR. We fitted a GLM to the counts for each transcript using a design matrix with a one-way layout. This was set up using two groups of induced or control cells for each 416B plate, and with one group containing all cells for each TSC plate. To simulate counts, we recalculated the fitted values of the GLM after setting the offsets of all cells to be equal to the mean offset. The cell- and transcript-specific fitted values and the transcript-specific dispersion were used as the parameters for a NB distribution from which counts were resampled. In this manner, simulated counts were obtained for each spike-in transcript in each cell.

Our aim is to compute the variance of the log-ratios of the total counts between spike-in sets from the simulated data. By forcing the offsets of all wells to be equal, we removed differences in the log-ratios between wells due to cell-specific capture efficiency and sequencing depth. This means that any variance in the simulated log-ratios across wells must be due to stochastic sampling noise. Estimates of this variance represent the approximate contribution of $\sigma^2_{lib(1)} + \sigma^2_{lib(2)}$ to $\text{var}(\theta^*_i)$ (Supplemental Figure 7, at 100% coverage).

We also downscaled the fitted values to determine the effect of reducing spike-in coverage on the sampling noise. Specifically, we repeated the simulations after scaling the fitted values down to 1% of their original values, to mimic the addition of less spike-in RNA to each well. To preserve the mean-variance relationship,

we fitted a loess curve of degree 1 to the original log-transformed NB dispersion against the log-mean of the fitted values across all cells. We used this trend to determine the new NB dispersion for each transcript after downscaling. Count sampling was then performed using the scaled fitted values and new dispersions.

We did not examine the effect of reducing spike-in coverage on σ^2_{vol} or $\text{var}(F_i)$. Both of these terms refer to experimental processes that should be independent of the concentration of spike-in RNA. For example, the process of adding a volume of a solution is unrelated to the solution's contents. Similarly, the process of capturing a transcript molecule is unrelated to the number of other transcript molecules, excepting the presence of limiting reagents. Thus, the true values of σ^2_{vol} or $\text{var}(F_i)$ should generally be unaffected by the depth of coverage. In contrast, sampling noise during sequencing depends on the coverage and will contribute to the true value of $\sigma^2_{lib(s)}$. This motivated our simulations to test the effect of reducing coverage on $\sigma^2_{lib(s)}$.

2.2 Calculating the effect of noise on size factor precision

The above simulations describe the contribution of stochastic noise to the log-ratios in our mixture experiments. However, we can also perform simulations to quantify the effect of noise on the precision of the spike-in size factors themselves. For a given data set, we first performed quality control by removing cells with small outlier values (i.e., more than 3 median absolute deviations below the median) for the log-transformed total endogenous counts or for the log-total spike-in counts. We estimated the NB dispersion and fitted a GLM to the spike-in counts for each transcript, using the log-sum of spike-in counts as the GLM offset for each cell. For each transcript in each cell, we used the corresponding fitted value and dispersion estimate to sample a new count from a negative binomial distribution, yielding a new set of simulated spike-in counts.

To estimate a size factor for each cell, we calculated the sum of simulated counts for all spike-in transcripts in each cell. The count sums were scaled so that the average value across all cells in the data set was equal to unity. The scaled count sum for each cell was then used as its spike-in size factor. We repeated this procedure with a new set of simulated counts for multiple iterations, and we defined the estimation error as the standard deviation of the estimated size factors for the same cell across iterations. This was expressed as a percentage of the original value of the size factor (i.e., computed from the original counts) for each cell. We performed these simulations on the data sets in Section 4 to obtain Supplementary Figure 8.

We stress that these simulations only examine the variability in spike-in counts due to random sampling noise. It is not possible to make any precise conclusions regarding variability in the *amount* of spike-in RNA added to each cell. To do so requires the use of spike-in mixtures, which are not available in public data.

3 Examining index switching on the HiSeq 4000

Cells from three of our plates were multiplexed and sequenced on the Illumina HiSeq 4000 machine using the ExAmp chemistry. A recent study showed that the index primers used for barcoding each sample could switch between samples (Sinha et al., 2017), thereby contaminating one sample with DNA from other samples. We examined the effect of index switching on our 416B data, exploiting the fact that the first plate was generated on the HiSeq 2500 while the second was generated on the HiSeq 4000. The variance estimates were very similar between the two plates (Figure 2), suggesting that index switching has minimal effect on our conclusions. We also made use of the presence of the *CBFB-MYH11* oncogene, the expression of which was upregulated in half the cells on each plate. We observed similar log-fold changes between induced and control cells for both plates (Supplementary Figure 13a, b) whereas we would have expected that the log-fold change for the second plate would be closer to zero if samples were homogenized due to index switching.

For the TSC data set, each plate contained a negative control well where no cell was added. Any counts for endogenous mouse genes in those control wells could be attributed to index switching (or to other sources of contamination, e.g., cell-free RNA). In each plate, the total count across all mouse genes for each negative control well was around 2-3 orders of magnitude lower than the total mouse counts in the majority of wells (Supplementary Figure 13c, d). This suggests that, at worst, index switching would result in 1% of the library size for each well being attributable to contamination from other wells. To put this result into context, the coefficients of variation for the total ERCC count in the first and second plate are 0.29 and 0.26, respectively. This means the coverage routinely varies by 26-29% across wells due to cell-specific biases. By comparison, the 1% (maximum) rate of contamination due to index switching is likely to have negligible effect.

4 Overview of data sets used in simulations

We obtained the following public data sets, containing counts for ERCC spike-in transcripts:

- Liver cells from Scialdone et al. (2015), obtained from the ArrayExpress repository with the accession E-MTAB-3707. No experimental factors were considered.
- Brain cells from Zeisel et al. (2015), obtained from http://linnarssonlab.org/cortex. Only the subset of cells from the cortex was used. No experimental factors were considered.
- Mouse embryonic stem cells from Buettner et al. (2015), obtained from ArrayExpress with the accession E-MTAB-2805. Cell cycle phase (G1, G2M or S) was treated as the experimental factor.
- Mouse embryonic stem cells from Kolodziejczyk et al. (2015), obtained from http://www.ebi.ac.uk/teichmann-srv/espresso. The culture condition (2i, a2i or lif) was treated as the experimental factor. Only the subset of cells from batch 3 was used as these contained spike-ins for all conditions.
- Mouse embryonic stem cells from Islam et al. (2014), obtained from the Gene Expression Omnibus (GEO) with the accession GSE46980. No experimental factors were considered.
- Mouse embryonic stem cells from Grun et al. (2014), obtained from GEO with the accession GSE54695. The culture condition (2i or serum) was treated as a experimental factor.
- Fibroblasts from Hashimshony et al. (2016), obtained from GEO with the accession GSE78779. No experimental factors were considered. Only the subset of cells from the C1 experiment was used.
- Pancreatic islet cells from Segerstolpe et al. (2016), obtained from ArrayExpress with the accession E-MTAB-5061. Cells annotated as low quality were removed. (For downstream analyses, we considered all remaining cells as high-quality, so no further cell-level quality control was applied.) We only used the cells extracted from a single individual (HP1502401, healthy male) for simplicity.

In each of our TSC and 416B data sets, data from both plates were analyzed together. We treated the plate of origin for each cell as an experimental factor, along with the oncogene induction status for 416B cells. Only the ERCC counts were used in simulations, and all SIRV transcripts were discarded for simplicity.

5 Implementation details for the downstream analyses

5.1 Quality control on cells and genes

Cell-level quality control was performed on each data set by removing cells with outlier values for various metrics (Lun et al., 2016). These metrics included the log-transformed total read count across all genes and the log-transformed total number of expressed genes, where small outliers were removed; and the proportion of reads mapped to spike-in transcripts, where large outliers were removed. Outliers were defined as values that were more than three median absolute deviations away from the median in the specified direction. Genes were also removed if the average count across all cells was below 0.1. This reduces computational work by filtering out low-abundance genes that do not contain enough information for reliable inference.

5.2 Methods for detecting differentially expressed genes

For DEG detection with edgeR v3.18.1, a NB GLM was fitted to the counts for each gene (McCarthy et al., 2012) using a suitable design matrix. The design matrix for each data set was constructed using an additive parameterization for all experimental factors, including the factor containing the conditions to be compared for differential expression. The log-transformed total count for the spike-in transcripts was used as the offset for each library. This is equivalent to spike-in normalization as each count is effectively downscaled by the spike-in total. An abundance-dependent trend was fitted to the NB dispersions of all genes using the estimateDisp function. For each gene, empirical Bayes shrinkage was performed towards the trend to obtain a shrunken NB dispersion, and the likelihood ratio test was applied to test for significant differences in expression between conditions. The Benjamini-Hochberg correction was applied to control the FDR.

For MAST v1.2.1, an effective library size was defined by multiplying the total spike-in count by a constant value C for each library. We set C to the ratio of the average total count for the endogenous genes to the average total count for the spike-in transcripts, where each average was computed across all libraries. This procedure recapitulates the effect of spike-in normalization by ensuring that the fold-differences in the effective library sizes are equal to the fold-differences in the spike-in totals between cells. Counts were converted to count-per-million (CPM) values using the effective library sizes, which were further log-transformed after adding a pseudo-count of 1. For each gene, a hurdle model was fitted to the log-CPMs across all cells using the zlm function with default parameters. This was parameterized as an additive model for all experimental factors, with the proportion of genes with non-zero counts in each library included as a covariate (Finak et al., 2015). Putative DE genes between the relevant conditions were identified using the lrTest function.

5.3 Methods for detecting highly variable genes

The first HVG detection method was based on the approach described by Brennecke et al. (2013), with some modifications to the size factors to perform spike-in normalization. Specifically, the spike-in totals were scaled so that their mean across all libraries was equal to unity. The size factor for each library was defined as its scaled spike-in total. We did *not* compute separate size factors for the endogenous genes and spike-in transcripts, as this would require the use of non-DE normalization methods. The rest of the method was implemented as originally described, using the technicalCV2 function in the scran package (v1.4.4) with min.bio.disp set to zero. If any experimental factors were present, they were regressed out by log-transforming the counts; applying the removeBatchEffect function from the limma package (Ritchie et al., 2015) to the log-counts; and converting the corrected log-values back to the count scale, prior to using technicalCV2.

The second approach to detect HVGs was based on computing the variance of log-expression values (Lun et al., 2016). For each count in each library, a normalized log-expression value was defined as the log-ratio of the count with the spike-in size factor for that library. (A pseudo-count of 1 was added to avoid undefined values upon log-transformation.) If any experimental factors were present, they were used to construct a design matrix with an additive parameterization – otherwise, an intercept-only design matrix was used. The design matrix was used to fit a linear model to the log-expression values to obtain the residual variance for each spike-in transcript. A loess curve was fitted to the log-variance against the mean for all spike-in transcripts using the trendVar function in scran, representing the mean-variance relationship due to technical noise. Variance estimation was repeated on the log-expression values for each endogenous gene, and the biological component of the variance and a p-value was computed for each gene using the decomposeVar function.

5.4 Methods for dimensionality reduction and clustering

Size factors for all libraries were defined from the spike-in totals as previously described. HVG detection was performed using the variance-of-log-expression method, where HVGs were defined as genes detected at a FDR of 5% and with biological components above 0.5. PCA was performed on the normalized log-expression values of the HVGs, using the prcomp function from the stats package with scaling. The first two PCs were used as the coordinates for each cell in one PCA plot, and the first and third PCs were used as coordinates in another plot. Each point was coloured according to its annotated cell type (Segerstolpe et al., 2016).

The procedure above was repeated at each simulation iteration with new spike-in counts. Coordinates of all cells in each simulated PCA plot were mapped onto the original plot, after scaling and rotating the coordaintes to eliminate differences between plots that were not relevant to interpretation. Specifically:

- For each cell, the simulated coordinates were right-multiplied by a 2-by-2 transformation matrix

$$\begin{bmatrix} b_x \cos(\psi) & -b_y \sin(\psi) \\ b_x \sin(\psi) & b_y \cos(\psi) \end{bmatrix}$$

  where ψ is the angle around the origin and b_x and b_y scale the x- and y-coordinates, respectively.

- The squared Euclidean distance from the (scaled and rotated) simulated coordinates to the original coordinates was computed for each cell, and summed across all cells.

- The scaling and rotation parameters of the matrix were identified that minimized the sum of squared distances across all cells, using the optim function from the stats package.

The more obvious approach to remapping is to directly project the simulated log-expression data onto the space of the original plot. However, we do not do this as it does not capture the variability in the identification of the PCs across iterations. Upon completion of the simulation, each cell will have one original location and one remapped location per iteration. For each cell, the smallest circle centered at its original location was drawn that contained 95% of the remapped locations. This avoids inflated circles due to outliers.

References

Brennecke P, Anders S, Kim JK, Kolodziejczyk AA, Zhang X, Proserpio V, Baying B, Benes V, Teichmann SA, Marioni JC, et al. 2013. Accounting for technical noise in single-cell RNA-seq experiments. *Nat Methods* **10**(11):1093–1095.

Buettner F, Natarajan KN, Casale FP, Proserpio V, Scialdone A, Theis FJ, Teichmann SA, Marioni JC, Stegle O. 2015. Computational analysis of cell-to-cell heterogeneity in single-cell RNA-sequencing data reveals hidden subpopulations of cells. *Nat. Biotechnol.* **33**(2):155–160.

Finak G, McDavid A, Yajima M, Deng J, Gersuk V, Shalek AK, Slichter CK, Miller HW, McElrath MJ, Prlic M, et al. 2015. MAST: a flexible statistical framework for assessing transcriptional changes and characterizing heterogeneity in single-cell RNA sequencing data. *Genome Biol* **16**:278.

Grun D, Kester L, Oudenaarden A van. 2014. Validation of noise models for single-cell transcriptomics. *Nat. Methods* **11**(6):637–640.

Hashimshony T, Senderovich N, Avital G, Klochendler A, Leeuw Y de, Anavy L, Gennert D, Li S, Livak KJ, Rozenblatt-Rosen O, et al. 2016. CEL-Seq2: sensitive highly-multiplexed single-cell RNA-Seq. *Genome Biol* **17**:77.

Islam S, Zeisel A, Joost S, La Manno G, Zajac P, Kasper M, Lonnerberg P, Linnarsson S. 2014. Quantitative single-cell RNA-seq with unique molecular identifiers. *Nat Methods* **11**(2):163–166.

Kolodziejczyk AA, Kim JK, Tsang JC, Ilicic T, Henriksson J, Natarajan KN, Tuck AC, Gao X, Buhler M, Liu P, et al. 2015. Single cell RNA-sequencing of pluripotent states unlocks modular transcriptional variation. *Cell Stem Cell* **17**(4):471–485.

Law CW, Chen Y, Shi W, Smyth GK. 2014. voom: Precision weights unlock linear model analysis tools for RNA-seq read counts. *Genome Biol* **15**(2):R29.

Lun AT, McCarthy DJ, Marioni JC. 2016. A step-by-step workflow for low-level analysis of single-cell RNA-seq data with Bioconductor. *F1000Res* **5**:2122.

McCarthy DJ, Chen Y, Smyth GK. 2012. Differential expression analysis of multifactor RNA-Seq experiments with respect to biological variation. *Nucleic Acids Res* **40**(10):4288–4297.

Ritchie ME, Phipson B, Wu D, Hu Y, Law CW, Shi W, Smyth GK. 2015. limma powers differential expression analyses for RNA-sequencing and microarray studies. *Nucleic Acids Res* **43**(7):e47.

Scialdone A, Natarajan KN, Saraiva LR, Proserpio V, Teichmann SA, Stegle O, Marioni JC, Buettner F. 2015. Computational assignment of cell-cycle stage from single-cell transcriptome data. *Methods* **85**:54–61.

Segerstolpe A, Palasantza A, Eliasson P, Andersson EM, Andreasson AC, Sun X, Picelli S, Sabirsh A, Clausen M, Bjursell MK, et al. 2016. Single-cell transcriptome profiling of human pancreatic islets in health and type 2 diabetes. *Cell Metab* **24**(4):593–607.

Sinha R, Stanley G, Gulati GS, Ezran C, Travaglini KJ, Wei E, Chan CKF, Nabhan AN, Su T, Morganti RM, et al. 2017. Index switching causes "spreading-of-signal" among multiplexed samples in Illumina HiSeq 4000 DNA sequencing. *bioRxiv.* DOI: 10.1101/125724.

Zeisel A, Munoz-Manchado AB, Codeluppi S, Lonnerberg P, La Manno G, Jureus A, Marques S, Munguba H, He L, Betsholtz C, et al. 2015. Brain structure. Cell types in the mouse cortex and hippocampus revealed by single-cell RNA-seq. *Science* **347**(6226):1138–1142.

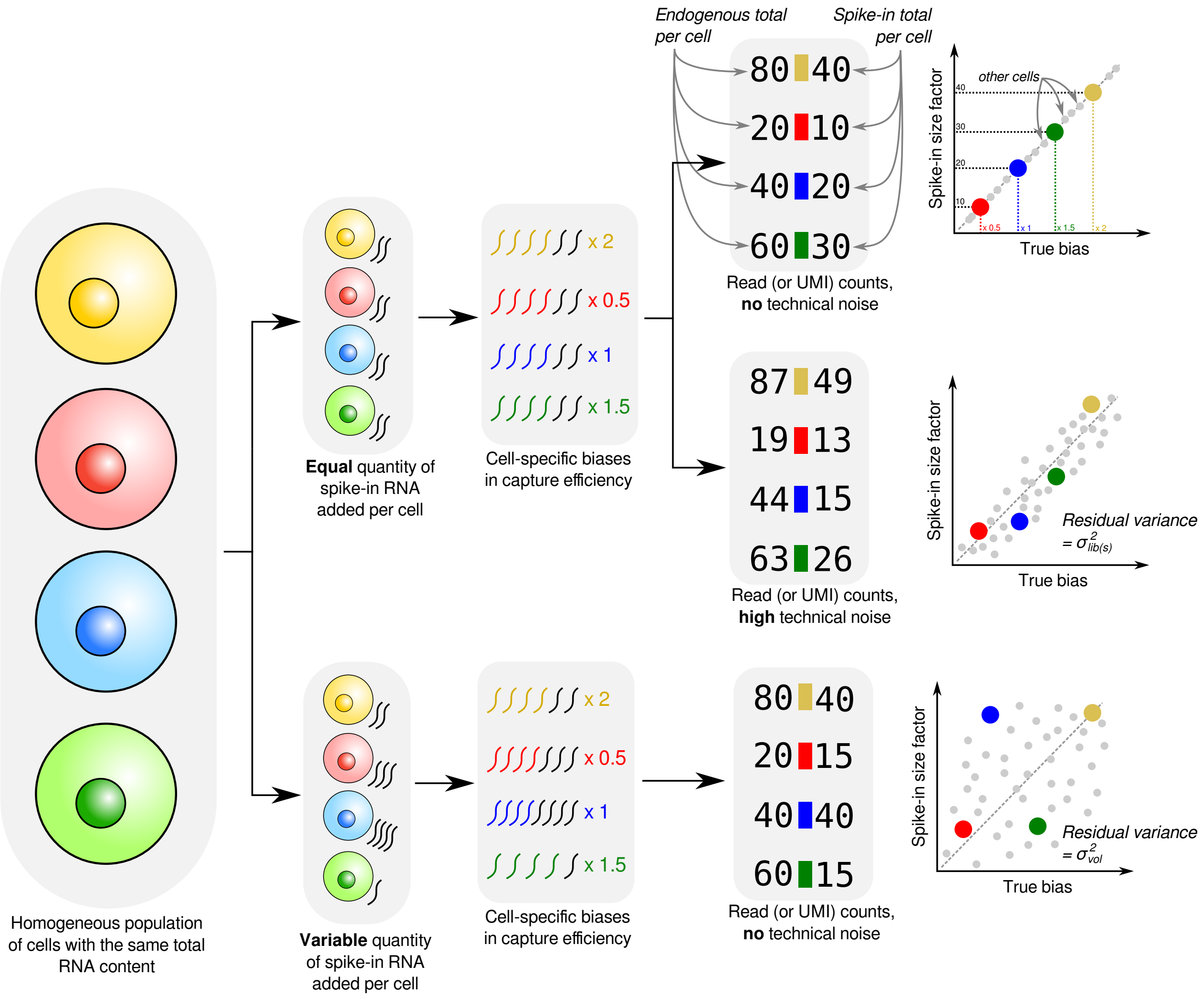

Supplemental Figure 1: Schematic of the effect of variability in spike-in addition (σ^2_{vol}) or library preparation ($\sigma^2_{lib(s)}$) on the performance of spike-in normalization. The spike-in size factor for each cell is calculated from the sum of counts for spike-in transcripts. The size factors faithfully represent the true cell-specific biases when equal amounts of spike-in RNA are added to each well and technical noise in library preparation and sequencing is low. This is not true when spike-in addition is variable or technical noise is high, manifesting as errors relative to the true biases (i.e., large residual variance). Note that the true biases are unknown, so σ^2_{vol} and $\sigma^2_{lib(s)}$ are instead estimated from spike-in mixtures as described in our experimental design.

Supplemental Table 1: Alignment and counting statistics for each batch of scRNA-seq data, including the total number of fragments (reads for single-end data, read pairs for paired-end data), percentage of reads mapped to the reference genome and percentage of fragments assigned to genic regions. For each statistic, the median value across all wells in the batch is shown with first and third quartiles in brackets.

**Data set**	**Total ($\times 10^6$)**	**Mapped (%)**	**Counted (%)**
416B (I)	2.80 (2.39-3.10)	59.2 (56.6-61.6)	46.3 (45.1-49.3)
416B (II)	2.82 (2.40-3.26)	50.3 (47.3-53.1)	39.0 (36.5-42.3)
Tropho (I)	2.02 (17.9-2.20)	88.8 (88.1-89.3)	74.9 (73.0-76.5)
Tropho (II)	2.33 (2.08-2.57)	89.1 (87.6-89.7)	62.8 (61.5-65.5)

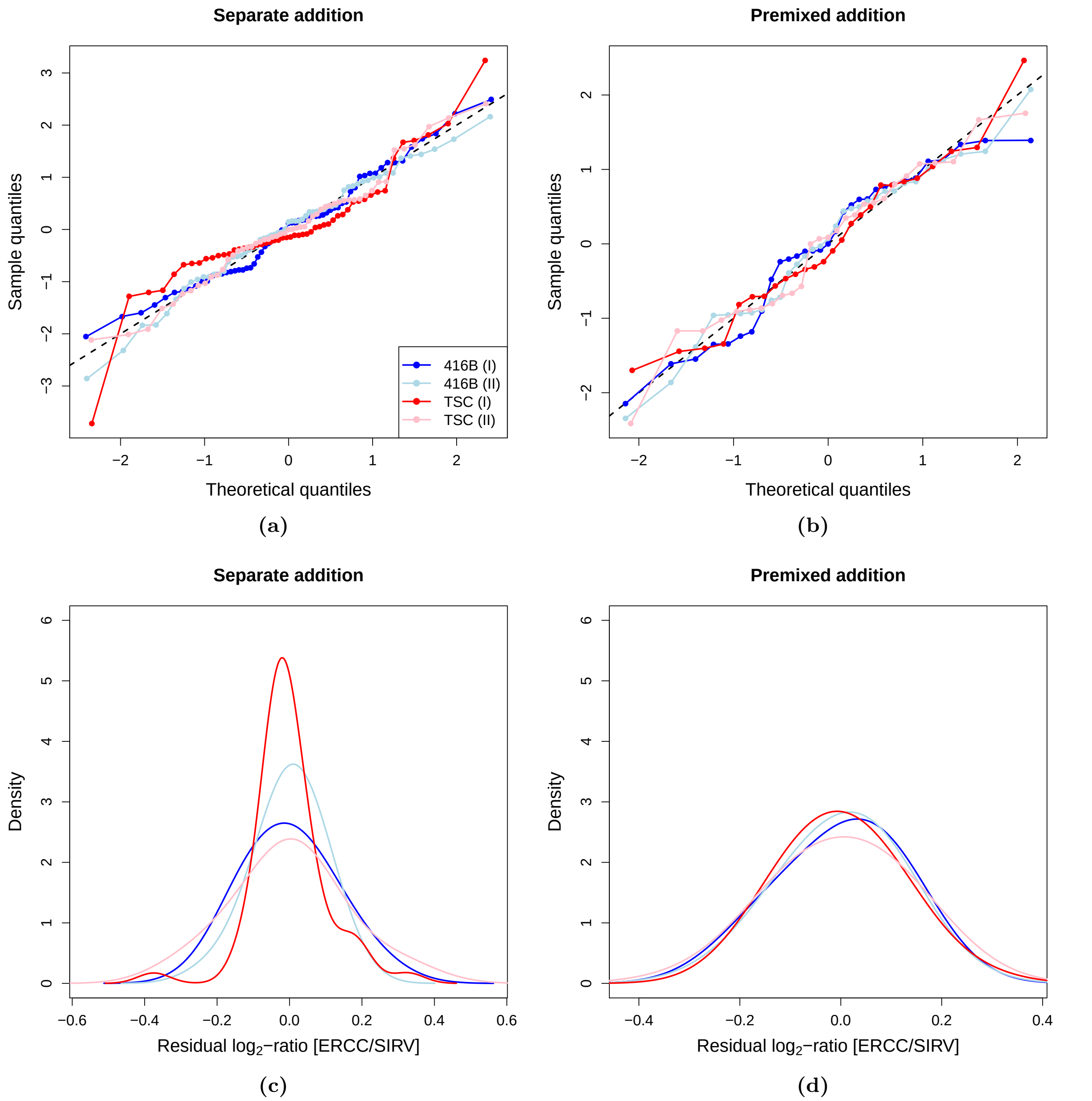

Supplemental Figure 2: Distribution of the log-ratios after separate or premixed addition of spike-in sets. For each plate, a linear model was fitted to the log-ratios of the corresponding wells to account for factors in the experimental design. (a, b) Quantile-quantile plots of the residuals of the fitted model for each plate. Residuals were standardized and plotted against the theoretical quantiles of a standard normal distribution. The dotted line represents equality between the sample and theoretical quantiles. (c, d) Density estimates of the residuals from each plate, computed using a Gaussian kernel with twice the default bandwidth.

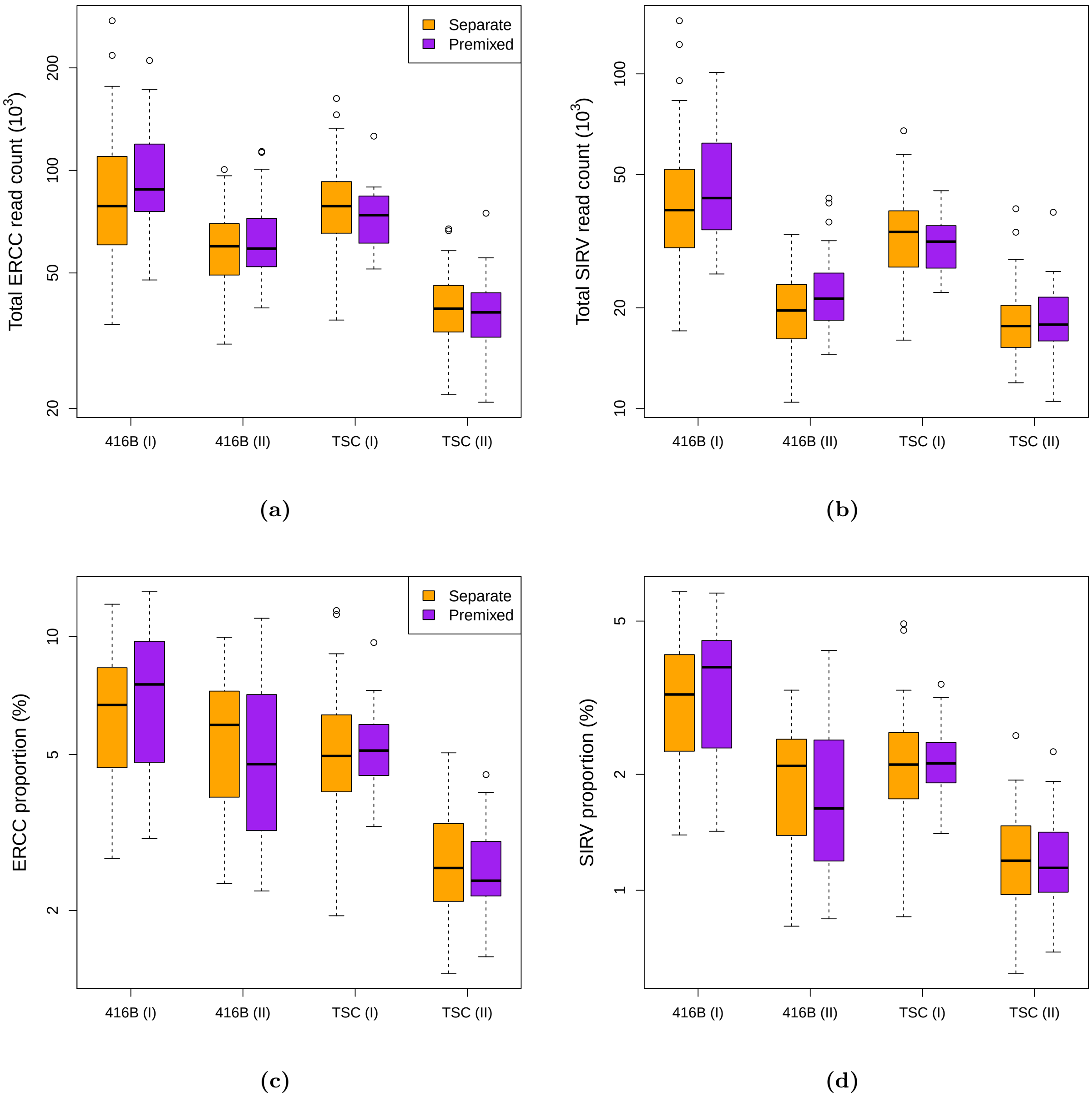

Supplemental Figure 3: Distribution of the coverage of the (a, c) ERCC or (b, d) SIRV spike-in set across wells, shown as the total number of reads assigned to the spike-in transcripts (a, b) or as a proportion of the total number of reads in the corresponding library (c, d). For each plate, separate boxplots are shown for wells in which spike-ins were added separately or premixed before addition. Dots represent wells that are more than 1.5 interquartile ranges from the first or third quartile of the corresponding distribution.

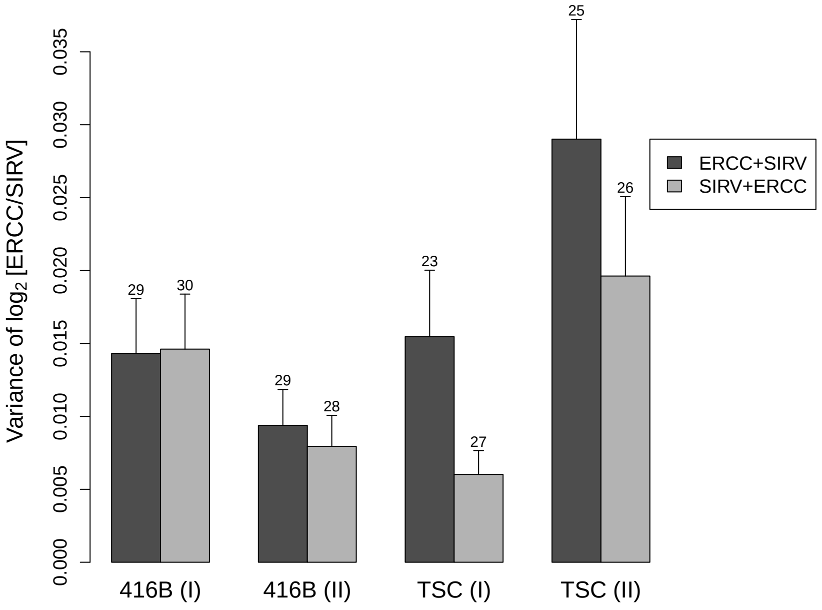

Supplemental Figure 4: Estimated variance of the log-ratio of total counts between spike-in sets, computed across wells in which the ERCC spike-in set was added before the SIRV set or vice versa. This exploits the presence of a number of wells in each plate (indicated above each bar) for which the order of spike-in addition was reversed. Error bars represent standard errors of the variance estimates for normally distributed log-ratios. Differences between the ERCC- and SIRV-first estimates of each batch were assessed using a two-sided F-test, yielding p-values of 0.96, 0.66, 0.02 and 0.33 for the respective batches from left to right.

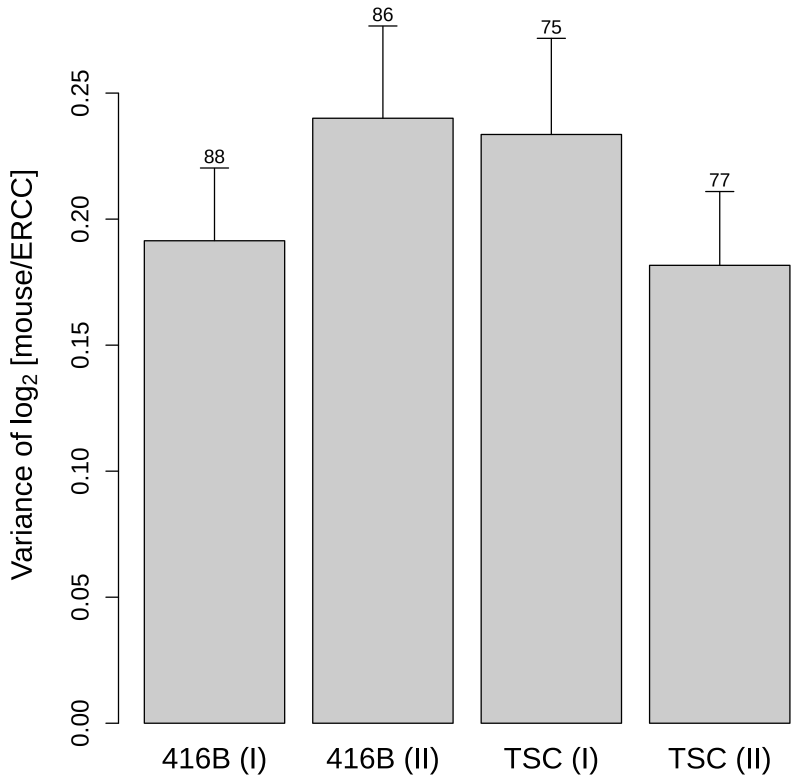

Supplemental Figure 5: Estimated variance of the log-ratio of total counts across all endogenous mouse genes to that across all ERCC transcripts, computed across all wells on each plate (numbers shown above each bar). Error bars represent standard errors of the variance estimates for normally distributed log-ratios.

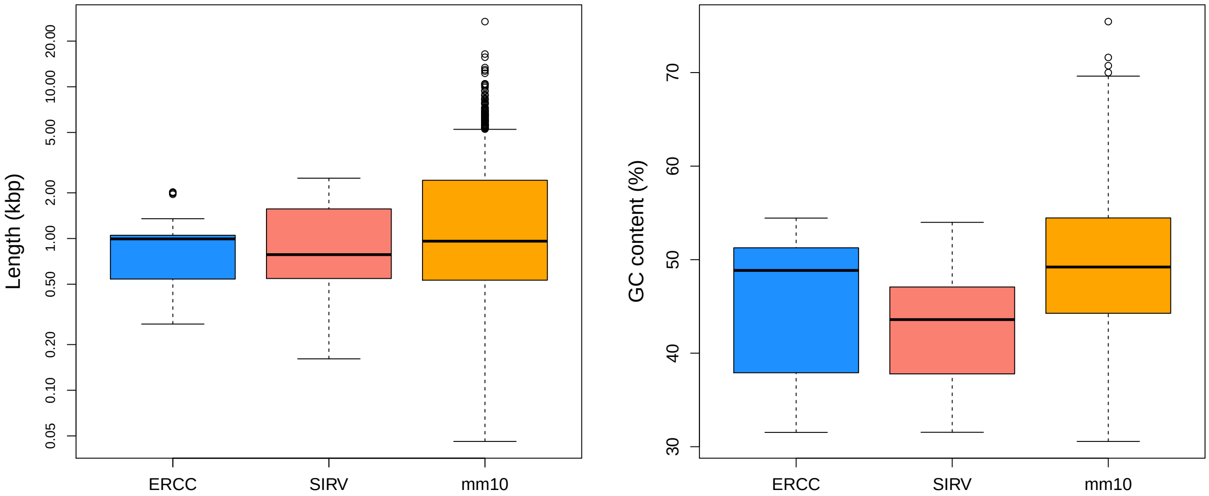

Supplemental Figure 6: Biophysical properties of transcripts in each of the two spike-in sets and for 2000 randomly selected transcripts from the mouse mm10 genome. Boxplots are shown for the distribution of lengths and GC contents of transcripts (not including the poly-A tail) in each set.

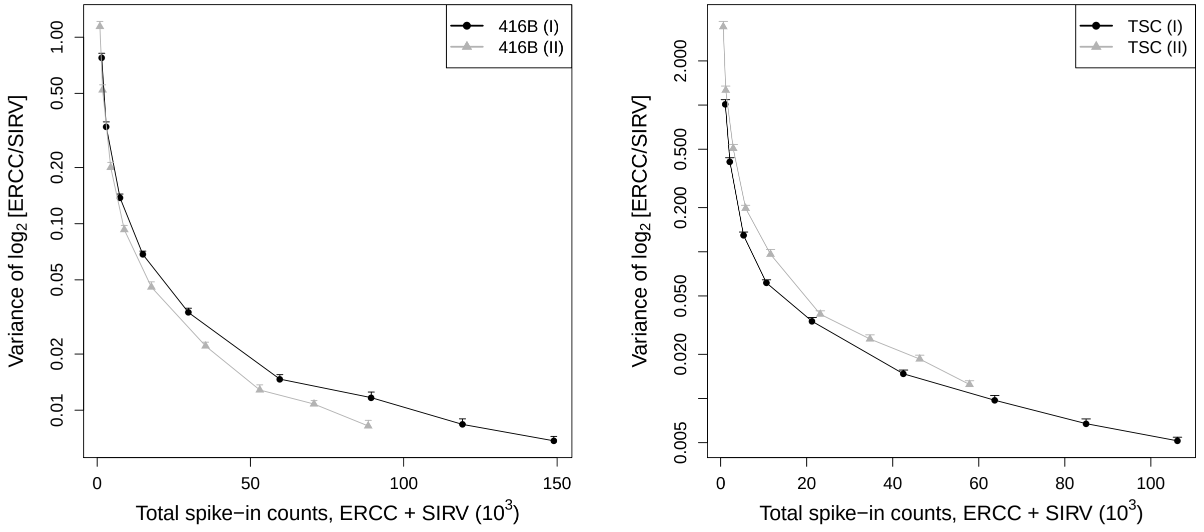

(a) **(b)**

Supplemental Figure 7: Effect of spike-in coverage on the variance of the log-ratios of the ERCC and SIRV total counts, using simulations based on the (a) 416B or (b) TSC data sets. Simulations were performed where the only source of variation between cells was the stochastic sampling of counts from a NB distribution with parameters estimated from real data. This was repeated after scaling the mean count for each spike-in transcript to 1-100% of the original coverage. The last point of each line represents the variance at 100% coverage on the corresponding plate. The total count of both spike-in sets was computed as an average across all cells on the plate, and is used here to quantify overall coverage of the spike-ins after scaling. (See Supplemental Figure 3 for the original total count of each set.) Each point represents the mean variance from 20 simulation iterations, with error bars representing standard errors of the variance estimates.

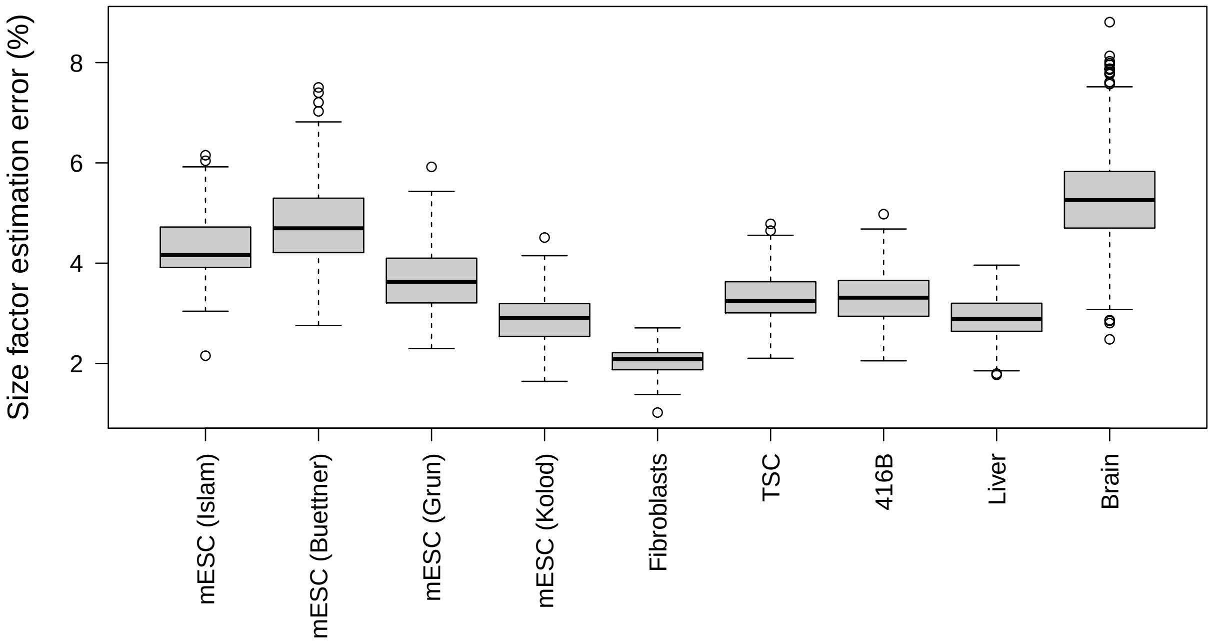

Supplemental Figure 8: Relative estimation error for the spike-in size factor of each cell, shown as a percentage of the value of the size factor. Boxplots represent the distribution of errors across cells in each data set. Errors were calculated after resampling spike-in counts from a negative binomial distribution with parameters estimated from the original counts. Calculations were performed using data for mouse embryonic stem cells (mESCs) from Islam et al. (2014), Buettner et al. (2015), Grun et al. (2014) and Kolodziejczyk et al. (2015); fibroblasts from Hashimshony et al. (2016); trophoblast stem cells (TSCs) and 416B cells from our experiments; liver cells from Scialdone et al. (2015); and brain cells from Zeisel et al. (2015).

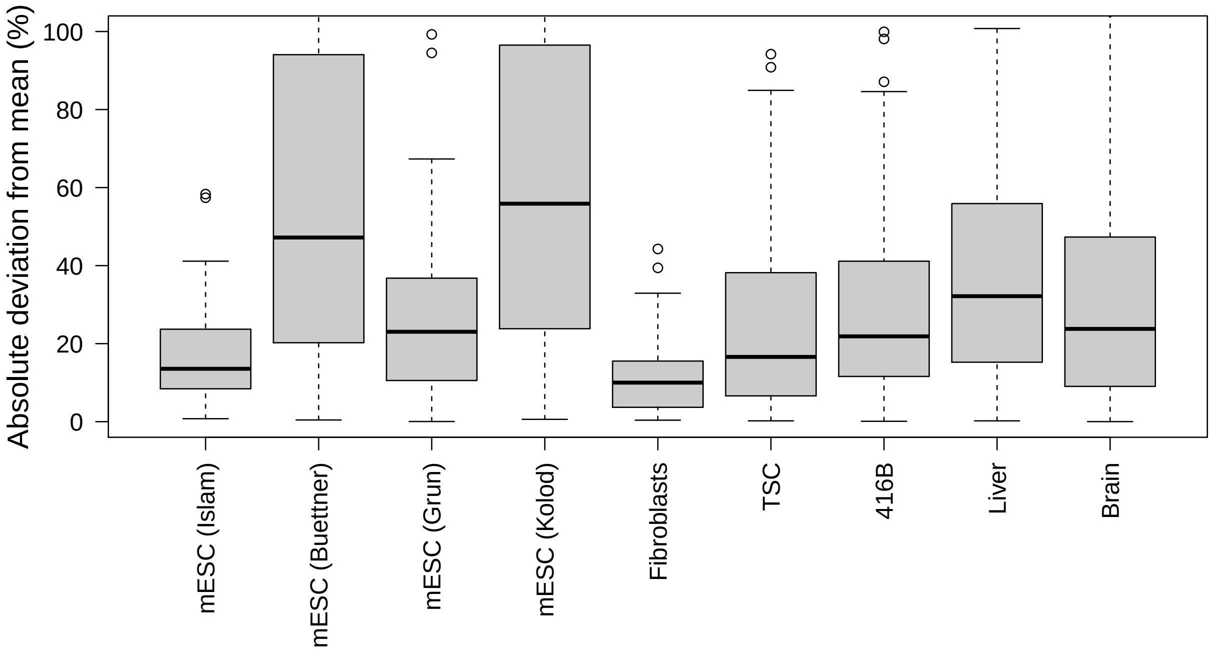

Supplemental Figure 9: Absolute deviation of the spike-in size factor of each cell, shown as a percentage of the mean size factor across cells. Deviations were calculated by fitting a linear model to the $\log_2$-size factors, using a design matrix containing all experimental factors in the data set. The deviation for each cell was defined as $|2^r - 1| \times 100$ where r is the corresponding residual for that cell in the fitted model. Each boxplot represents the distribution of deviations across cells in a particular data set, see Supplemental Figure 8.

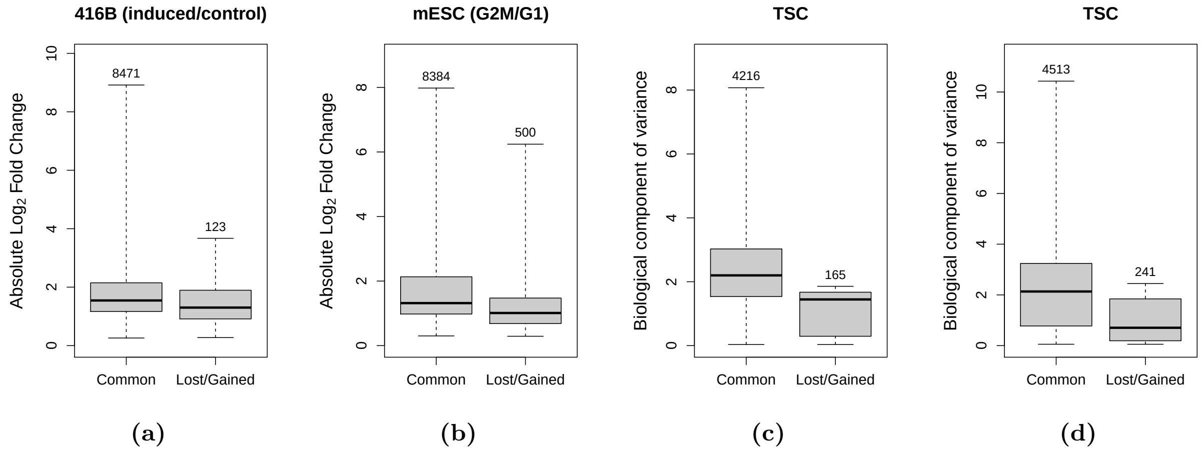

Supplemental Figure 10: Properties of genes that were detected as DEGs or HVGs in both the original and simulated data with spike-in variability (common), compared to genes that were no longer detected or additionally detected after simulation (lost/gained). (a, b) Absolute magnitudes of the log-fold changes of DEGs from edgeR in the 416B or mESC cell cycle data sets. (c, d) Biological components of variation of HVGs from the variance of log-expression method in the 416B and TSC data sets. The number of genes in each category is also shown for each data set. Similar results were obtained for the CV^2 method and MAST.

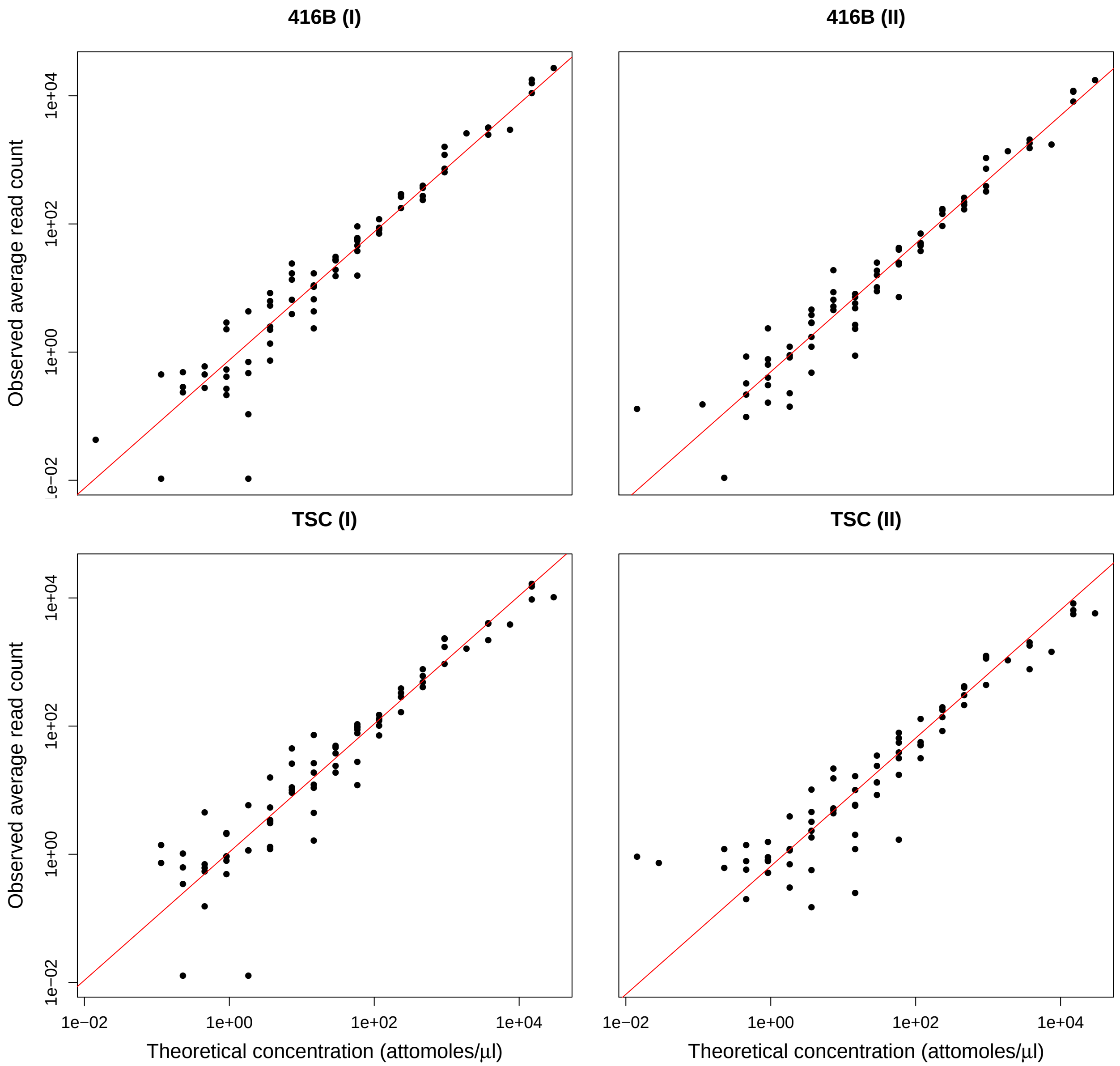

Supplemental Figure 11: Observed average read count for each transcript in the ERCC spike-in set, plotted against the theoretical concentrations (obtained as Mix A in "ERCC Controls Analysis: ERCC RNA Spike-In Control Mixes (English)" at https://www.thermofisher.com/order/catalog/product/4456740). Each point represents a spike-in transcript, with counts averaged across all wells in a plate. The red line represents a line of best fit with gradient 1. Equivalent plots for the SIRVs are not shown as the relationship between the observed count and the theoretical molar concentration is not straightforward in the presence of isoforms.

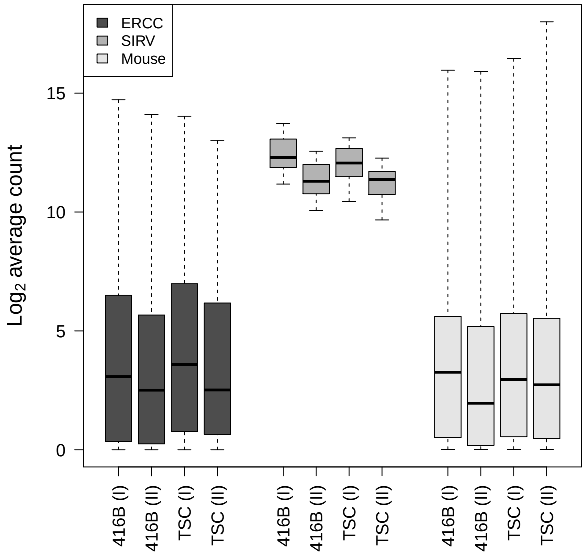

Supplemental Figure 12: Distribution of average counts across the ERCC and SIRV spike-in transcripts and endogenous mouse genes. For each feature, counts were averaged across all wells on each plate.

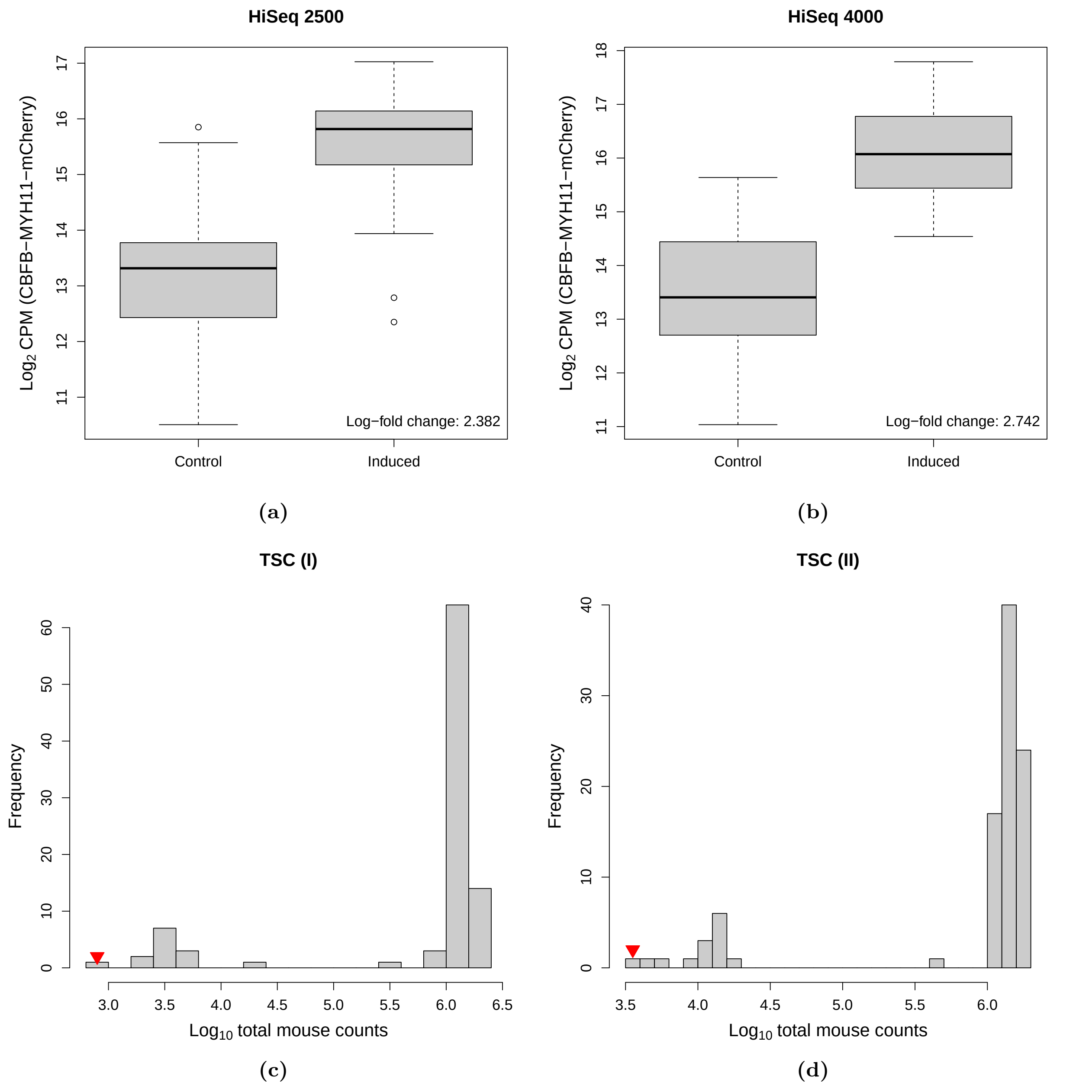

Supplemental Figure 13: Effect of index switching in each data set for the mixture experiments. (a, b) Log-fold change of *CBFB-MYH11* oncogene expression in control and induced 416B cells on each plate, generated using the HiSeq 2500 (plate I) or 4000 (II). (c, d) Distribution of log-total counts for mouse genes for all wells on each TSC plate. The negative control well on each plate is marked by the red triangle.
